# Computable Features Required to Evaluate the Efficacy of Drugs and a Universal Algorithm to Find Optimally Effective Drug in a Drug Complex

**DOI:** 10.1371/journal.pone.0033709

**Published:** 2012-03-23

**Authors:** Kui Wang, Wei Cui, Gang Hu, Jianzhao Gao, Zhonghua Wu, Xingye Qiu, Jishou Ruan, Yi Feng, Zhi Qi, Yiming Shao, Jack A. Tuszynski

**Affiliations:** 1 College of Mathematical Sciences and LPMC, Nankai University, Tianjin, People's Republic of China; 2 National Center for AIDS/STD Control and Prevention, Chinese Center for Disease Control and Prevention, Beijing, People's Republic of China; 3 National Key Laboratory for Pharmaceutical chemistry and biology at Nankai University, Tianjin, People's Republic of China; 4 Department of Physics, University of Alberta, Edmonton, Alberta, Canada; 5 Department of Oncology, University of Alberta, Edmonton, Alberta, Canada; Semmelweis University, Hungary

## Abstract

**Background:**

The H1N1 pandemic in 2009 and the H5N1 pandemic in 2005 demonstrated that the drugs approved to treat influenza A viruses have low efficacy. This provided a stimulus for new studies of influenza A viruses in the context of the methods used in drug design developed over the past 100 years. Finding new universal drugs is the ultimate goal but its long time horizon is incompatible with emergency situations created by reoccurring influenza outbreaks. Therefore, we propose a computer-aided method for finding efficacious drugs and drug complexes based on the use of the DrugBank database.

**Methods:**

(1) We start by assembling a panel of target proteins. (2) We then assemble a panel of drugs. (3) This is followed by a selection of benchmark binding pockets based on the panel of target proteins and the panel of drugs. (4) We generate a set of computational features, which measure the efficacy of a drug. (5) We propose a universal program to search for drugs and drug complexes. (6) A case study we report here illustrates how to use this universal program for finding an optimal drug and a drug complex for a given target. (7) Validation of the Azirchromycin and Aspirin complex is provided mathematically. (8) Finally, we propose a simple strategy to validate our computational prediction that the Azirchromycin and Aspirin complex should prove clinically effective.

**Result:**

A set of computable features are mined and then based on these features, a universal program for finding the potential drug &drug complexes is proposed. Using this universal program, the Azirchromycin and Aspirin complex is selected and its efficacy is predicted mathematically. For clinical validation of this finding, future work is still required.

## Introduction

### 1 General background

The H5N1 pandemic in 2005 and the H1N1pandemic in 2009 demonstrated the fact that there are no effective drugs to specifically treat infections caused by the Influenza A virus. Consequently, there is renewed interest in the studies of influenza A viruses. The recent discovery of CR6261 published by Throsby et al ([1], PLoS 2008), the spatial structure of HA binding with CR6261 (3gbn) published by Ekiert et al ([2], Science 2009) and the family of CR6261-like antibodies published by Sui et al ([3], Nat. Str. & Mol. Bio. 2009) provided a solid basis for future work on new vaccines. The first novel insight is the concept of universal vaccines proposed by Nabel et al ([4], Nature Medicine 2010) and Wei et al ([5], Science 2010). Following the prophylactic and therapeutic tests of the efficacy of CR6261 by Friesen et al ([6], PLoS 2010), they subsequently offered an insight suggesting to use CR6261 in combination with drug such as oseltamivir or zanamivir in order to effectively protect against all seasonal influenza viruses. These two recent insights provide a conceptual basis for new studies of influenza A viruses.

Inspired by the second insight, we propose to find a new drug with drug complexes, which would respond to emergency situations by repurposing old drugs rather than designing new ones from scratch. For simplicity, henceforth we will use the term “drug & drug” to represent “a combination of a drug with another drug”. In order to be able to accomplish this we need to determine the following: (1) what drugs are presently approved to treat influenza A viruses? (2) What proteins are the potential targets of these drugs? (3) What pockets are the binding sites for these drugs? (4) What factors were ignored when these approved drugs were designed?

The outline of this paper is as follows:

1. First, we assemble a panel of target proteins. It contains all potential proteins having either complete or partial 3D structures. While each drug has an assumed target protein (referred to as the benchmark protein), it is not always guaranteed that this is the actual benchmark protein. Using a panel of target proteins, we may be able to verify if a given protein is the benchmark protein for a given drug.
2. We then assemble a panel of drugs. To confirm that a drug is effective in treating influenza A, we should compare it with other effective drugs and other non-influenza viral drugs. Therefore, we assemble the known influenza viral drugs and the known non-influenza viral drugs to form a panel of drugs. Of course, we also ensure the uniformity of the sizes of the drug molecules when selecting the non-influenza viral drugs.
3. We next assemble a panel of benchmark pockets based on the panel of target proteins and the panel of drugs. At this point it is still unclear where on a protein surface the binding site is located (referred to as the benchmark pocket) for a given drug even if we know that the given protein is the benchmark protein for the drug. Therefore, determining the set of benchmark pockets is the only way to validate the efficacy of a drug or to design a new drug. We call this set the panel of benchmark pockets.
4. We then propose a set of computable features required to measure the efficacy of a drug. Each benchmark pocket has a specific spatial extent and performs a specific function. A drug (either already designed or yet to be designed) is just a ligand molecule that needs to fit into the spatial extent created by the pocket and to bind strongly enough to the target. Therefore, we should individually find computable features of each benchmark pocket as accurately as possible so that we can predict whether or not an approved drug (or a drug yet to be designed) satisfies these features.
5. Finally, we propose a universal program to find the potential drug & drug complexes. This is a very time-consuming process, which requires high performance computing resources.
6. We conclude by providing a case study that demonstrates that drug & drug complex (Azithromycin & Aspirin) can be found using this universal program. This drug & drug complex is predicted to be effective through mathematical analysis and computational algorithms.
7. We propose a simple and operable strategy to validate these predictions clinically.

### 2 What tools are used?

The first tool used in this paper is AutoDock software. The kernel index of AutoDock is the minimal free energy (MFE) which measures the fitness of a drug binding to a pocket. This involves geometrical features, chemical features and physical features [7]–[11]. The second tool is the Ligand Explorer which is an associated software package provided by DrugBank or Protein Data Bank (PDB). Using Ligand Explorer, we can fully understand the interactions between a drug and a binding pocket including the number of the non-covalent bonds and the distribution of the non-covalent bonds in the drug binding pocket. The third tool is molecular dynamics (MD) simulation software. MD simulation is regarded as a computational bridge between theory and experiment and between microscopic and macroscopic analyses. We refer the reader to relevant literature on the subject [12].

## Materials and Methods

### 1 The panel of target proteins

To design drugs or to evaluate the efficacy of an approved drug, we need to know either its complete 3D structure or at least a partially-determined spatial structure (e.g. a functional domain) of the target protein. Among all 11 influenza A viral proteins, only hemagglutinin (HA) and neuraminidase (NA) currently have complete 3D structures (i.e., 1rd8 and 2hu4). The partial structures (1nyj) of M2 have been known for a long time but a complete structure is still unknown. Partial spatial structures of trimeric polymerases (PA, PB1 and PB2) (i.e., 3hw3, 3hw4, 3hw5, 3hw6, 3cm8 and 2znl) have been published but their complete structures are still unknown. Therefore, the selection of target proteins to be used to design drugs is limited. Below we briefly describe these complete or partial 3D structures:

1. 1rd8 is the crystal structure of the uncleaved human H1 hemagglutinin from the extinct 1918 influenza virus published by Stevens et al in 2004 [13]. Of course, many versions of a complete spatial structure of HA observed in different subtypes (i.e., human H3, avian H5 and avian H7) have been submitted to the PDB database since HA is a unique target protein for the development of vaccines.
2. 2hu4 is a complete spatial structure of neuraminidase (NA) derived from the H5N1 avian influenza neuraminidase [14], but all subtypes in group N1 share the same complete spatial structure. The function of this protein is to release the N-acetylneuraminic acid in order to cleave HA so that viruses can escape from infected cells. Using NA as the target protein, two drugs Oseltamivir and Zanamivir were designed.
3. 1nyj is the spatial structure of the well-known M2-TMD (transmembrane tetrameric bundle) published by Nishimura et al in 2002 [15]. It represents an ion channel of a viral particle and therefore it is a traditional benchmark pocket. Targeting 1njy, the drug Amantadine was designed to prevent the germ cytoplasm inside the viral lipid envelope from jetting into the host cell to be replicated.
4. 3cm8 is the partial structure of the PA_C and PB1_N complex published by He et al ([16], Nature 2008). In the same volume of Nature in 2008, Obayashi et al [17] also published this partial structure named 2znl. Following from [16] we may find more details regarding the topological diagram of PA_C and the 3_10 helix formed by PB1_N, which is called eta2 and it corresponds to the 3_10 helix called eta1 on PA_C. The molecular basis for the interaction between the jaws and the tongue has been discussed in detail [18]. However, the interaction between eta1 and eta2 has not been elucidated.
5. 3hw3, 3hw4, 3hw5 and 3hw6 are four versions of the spatial structure of PA_N in complex with UMP, TMP, AMP and Mn, respectively, published jointly by Rao's and Liu's groups in 2009 [19]. These four versions become the same if we eliminate the substrates UMP, TMP, AMP and Mn. Therefore we select 3hw3 as the representative of the asymmetric unit. For 3hw3, we will find four identical chains (A, B, C and D) and each identical chain is just PA_N (1–196). These four chains form an asymmetric unit.

In summary, the information about the spatial structures of the influenza A viral proteins, is still very incomplete. Our panel of potential target proteins used to design drugs or vaccines only includes the following structures: 1rd8, 2hu4, 1nyj, 3cm8 and 3hw3.

### 2 The panel of drugs

In the past, Amantadine, Zanamivir and Oseltamivir were approved for the treatment of influenza A viruses. Nevertheless, the efficacies of these drugs insufficient to ensure that these drugs may cure either H1N1 or H5N1 infected patients. A popular explanation of this situation is that influenza A viruses mutate fast enough to develop drug resistance. This is equivalent to saying that influenza viruses have the intelligence that allows them to change themselves fast enough to make the drugs ineffective. We do not subscribe to this point of view since the mechanism of drug action of Amantadine is known and it involves the benchmark pocket, which is highly conserved in all subtypes. Furthermore, for Zanamivir and Oseltamivir, we can also find their benchmark pocket, which is highly conserved in all subtypes within group N1. Therefore, we should first analyze whether or not there is a hidden design flaw involving Amantadine, Zanamivir and Oseltamivir making these drugs ineffective. If a drug turns out to have no design flaw, then we can look for other causes of drug resistance. For this purpose, we need to construct a panel of drugs, which includes both influenza anti-virals and non-influenza anti-virals. In this paper we have selected nine drugs (i.e., Oseltamivir, Zanamivir, Amantadine, Aspirin, Azithromycin, Isosorbide, Vancomycin, Heroin and HEM) to form the panel of drugs under study. Oseltamivir, Zanamivir and Amantadine have been selected as influenza anti-virals (Friesen et al [6], Russell et al [13], Nishimura et al [15] and Wang et al [20]). The six non influenza viral drugs have been selected for comparison seems to be arbitrary. It is a longer story to get the panel. The details is shown in section 1 of Supporting Information S1, and we only show the weights, the target proteins and the groups of these drugs in Table 1 to tell readers this panel is balance and that 6 added drugs are really non influenza viral drugs in the sense of biomedical. In other word, the weights of three non influenza viral drugs approximate to that of three influenza viral drugs, while the weights of the other three non influenza viral drugs are strict larger.

**Table 1:** The weight and the target protein of the drugs in the panel.

drug	weight	Target protein	groups
Vancomycin	1449.254	1pnv	approved
Azithromycin	748.9845	50S ribosomal protein L4	approved
HEM	618.46	1bep	experimental
Heroin	369.411	Mu-type opioid receptor	illicit, experimental
Zanamivir	332.3098	NA (Neuraminidase)	approved
Ossltamivir	312.4045	NA (Neuraminidase)	approved
Isosorbide	191.1388	enzyme guanylate cyclase	approved
Aspirin	180.1574	COX-1/COX-2	approved
Amantadine	151.2487	Proton channel protein M2	approved

### 3 The panel of benchmark pockets

A drug's efficacy is contingent on its binding to a pocket of the target protein. Thus, for each drug we need to know the location of its benchmark pockets. Consequently, we need to know the 3D structure of the target protein and also a method is needed to explore the exact location of a pocket. AutoDock is a readily available and widely used software tool. On one occasion, we suspected either we had misused the AutoDock or AutoDcok has some major flaw because different drugs may be predicted to dock with the same place.

We first exclude that we had operated AutoDock wrongly because we had validated that Oseltamivir and Zanamivir can find their benchmark pocket on their target protein NA, that Amantadine can also find its benchmark pocket on its target protein M2, and that Fosamprenavir, Indinavir, Nelfinavir, Darunavir, Tipranavir and Amprenavir can also find their benchmark pocket on their target protein HIV-1 protease.

We also exclude that AutoDock has major flaw after we validated this result on a large panel of proteins and a large panel of ligands. In fact, selecting 1rd8, 2hu4, 1nyj, 3cm8, 3hw3, 1g6l, 2jle, 2gv9, 3gbn, 3gbm, 3fku, 3sdy, 3ztn and 3ztj as the target proteins, and choosing Amantadine, Aspirin, Azithromycin, HEM, Heroin, Isosorbide, Oseltamivir, Zanamivir and Vancomycin as the panel of drugs, then all of these ligands are predicted to be docked with the same pocket on each of above target proteins if these ligands can be packed into this pocket, while all of those ligands will arrive at the minimal value of minimal free energy at a neighborhood of the pocket if those ligands can not be packed into this pocket. Moreover, when the panel of ligands is enlarged to 34 ligands, these 34 drugs are also predicted to be docked with the same pocket uniformly. Furthermore, for the proteins formed by subunits (i.e, 3hw3, 3gbn, 3gbm, 3fku, 3sdy, 3ztn and 3ztj), if we just use a subunit as the target protein, then all ligands also are predicted to be docked with the same pocket on the subunit. Of course, this pocket on a subunit is not same as that pocket on entire protein. Especially, for the complete 3D-structures obtained from subtypes of human H3, avian H5 and avian H7, we repeat the same operations as for 1rd8, and find the same pocket in the similar location. For more detail, please see section 2 of Supporting Information S1.

**Figure 1:**
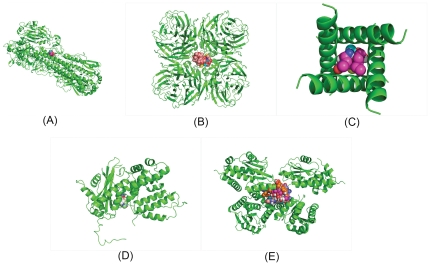
The pockets filled with drug&drug complex. (A).The position of pocket_1rd8 filled with Aspirin and Isosorbide.(B). The position of pocket_2hu4 filled with Aspirin, Isosorbide, Heroin, Oseltamivir and Azichromcin (C). The position of pocket_1nyj filled with Aspirin, Isosorbide, Heroin, Amantadine, Zanamivir and Oseltamivir. (D). The position of pocket_3cm8 filled with Aspirin, Isosorbide, Heroin, Amantadine, Zanamivir and Oseltamivir. (E). The position of pocket_3hw3 filled with all 9 drugs.

After above validation, not only we confidently trust that AutoDock is a reliable tool to find the benchmark pocket of drugs on the given target protein, but also we believe that AutoDock must contain a good preprocessing subprogram so that AutoDock always may escape from the trap of the locally minimal value so that all drugs may be sent to the same place to arrive at the global minimal value of MFE. Encouraged by this advantage, we have the idea to utilize this advantage sufficiently. In fact, if we input the 3D coordinates of a drug and the 3D coordinates of a protein, then AutoDock will outputs a value of the minimal free energy (MFE) and a predicted coordinates of the drug. Also, if we input a panel of drugs with the 3D coordinates and the 3D coordinates of a protein, then AutoDock will output a series of values of MFE and the predicted coordinates of the drugs. Therefore, if we show out all of these drugs with negative MFE using PyMOL according to the predicted coordinates at same time, then these drugs will be clustered in a void or a groove. And then we say this void/groove on a given target protein is a benchmark pocket of these drugs. In this way, we may know both the size and the location of the benchmark pocket. For example, we denote these five benchmark pockets on the target proteins 1rd8, 2hu4, 1nyj, 3cm8 and 3hw3 as pocket_1rd8, pocket_2hu4, pocket_1nyj, pocket_3cm8 and pocket_3hw3, respectively. We use orally-administered drugs for these benchmark pockets to show their binding locations (see Figure 1(A)–(E)).

Nevertheless, AutoDock still has a minor flaw. In practice, the predicted docking fashion may not be perfectly same as the real fashion observed using x-ray. For example, the complex structure of Indinavir docking with 2 bp× (one subtype of HVI-1 protease) obtained through x-ray is not perfectly same as the complex structure of Indinavir docking with 2 bp× predicted using Autodock, through both they are packed into the same benchmark pocket. Consequently, we should keep in mind that predicted efficacy of a dug may not be perfectly same as the real case. It is just a referential answer.

In the closing of this section, we state some information about the panel of benchmark pockets.

1. pocket_1rd8 is the smallest and its function is unclear. We may infer that it has no important function because the position of pocket_1rd8 is not close to the epitope on the spike to be docked by antibodies. Therefore, we ignore it.
2. pocket_2hu4 is mid-size and it represents the channel to release the N-acetylneuraminic acid (C_11_H_19_NO_9_). Importantly, it is highly conserved for these subtypes in group N1 and therefore it is used as the benchmark pocket of Oseltamivir and Zanamivir for preventing N-acetylneuraminic acid to be released. However, it was reported that both Oseltamivir and Zanamivir lead to drug resistance [11].
3. pocket_1nyj is the earliest known benchmark pocket. It is an ion channel. When ions pass through this channel and enter inside viral particles, they produce the pressure that allows the transfer of viral genetic material and proteins PA, PB1 and PB2 into human cells. Additionally, pocket_1nyj is highly conserved for all subtypes; hence it was used as the benchmark pocket to design the first groups of drugs. For example, the drug Amantadine was designed to block pocket_1nyj. Nevertheless, its clinical efficacy is not very impressive.
4. pocket_3cm8 is a new benchmark pocket and it is also highly conserved for all subtypes. However, its function is unclear (despite the known function of 3cm8 [7], [8]). We will not focus on this pocket in our paper.
5. pocket_3hw3 is the largest pocket of the set. Although 3hw3 is known as a cap-snitching domain and it is highly conserved, pocket_3hw3 has not been mentioned in the literature before now and its function is unclear. Despite our high degree of confidence in AutoDock, we still find it challenging to design a new drug that would be targeting it because we are familiar with any molecular mechanism associated with pocket_3hw3.

### 4. Mining the computable features to measure drug efficacy

#### 4.1. The minimal free energy (MFE)

The minimal free energy (MFE) is a well-known computable feature to describe compatibility between a drug and a benchmark pocket. Let us compare the values of MFE involving the nine drugs and five drug binding pockets obtained though the process of blind docking using AutoDock (see Table 2). In Table 2, the data underlined are positive and others are negative. From Figure 1(A)–(E), we find that the value of MFE is negative if the drug can be packed into the pocket, while the value of MFE is positive if the drug cannot be packed into the pocket. That is, the value of MFE is a good computable feature to measure the benchmark pocket whether or not it binds a drug.

**Table 2:** The values of MFE between 9 drugs and 5 drug binding pockets.

benchmark	Oseltamivir	Zanamivir	Amantadine	Aspirin	Azithromycin	Isosorbide	Vancomycin	Heroin	HEM
Pocket_1rd8	***51.85***	***71.83***	**−4.04**	**−3.09**	***4560***	**−3.6**	***19300***	***70.56***	***2360***
Pocket_2hu4	**−3.97**	**−7.43**	**−3.95**	**−3.64**	**−7.67**	**−3.85**	***4.95***	**−5.36**	**−6.56**
Pocket_1nyj	**−6.66**	**−7.14**	**−6.97**	**−5.16**	***4.52***	**−5.71**	***5970***	**−10.26**	***3.78***
Pocket_3cm8	**−5.07**	**−6.13**	**−5.22**	**−5.95**	***1380***	**−5.70**	***10100***	**−5.22**	***486.05***
Pocket_3hw3	**−3.07**	**−5.12**	**−3.82**	**−3.57**	**−5.46**	**−4.11**	**−7.30**	**−4.70**	**−4.97**

However, the value of MFE alone is not sufficient to evaluate the efficacy of a drug. In fact, if we only use the value of MFE as evidence, we will get many conflict implications. For example, we cannot distinguish the efficacies of Oseltamivir, Aspirin, and Isosorbide because their values of MFE are same level (see the row of pocket_2hu4 in Table 2), as well as, we cannot distinguish the efficacies of Zanamivir, Amantadine, Oseltamivir, Aspirin, Isosorbide and Herion because their values of MFE are almost in the same level (see the row of pocket_3cm8). Moreover, we will infer that Oseltamivir on pocket_1nyj is the best if we watch the column of Oseltamivir in Table 2, and we will also infer that Heroin on pocket_1nyj is best if we watch the row of pocket_1nyj in Table 2 since the value of MFE for Heroin is significantly larger than that value of MFE for Oseltamivir. Furthermore, we maybe infer that Vancomycin is much better than Oseltamivir based on the extreme values of MFE (−7.30 and −3.07) in the row of pocket_3hw3, which are significantly different because the std. error is about 2.33 [11]. However, the ordinary knowledge tells us that all 9 drugs are not effective on pocket_3hw3. Therefore, the value of MFE is not sufficient to evaluate the efficacy of a drug. We need more computable features to describe the efficacy of drugs.

#### 4.2. Two computable features related to the binding force

The binding force between a drug and a pocket, denoted by F, is an essential feature. Roughly, F is proportional to the number of non-covalent bonds if we ignore the angle. However, the simplified estimate of F is not sufficient to determine the direct fit between a drug and a pocket. Metaphorically, if we think of a pocket as the doorframe and a drug as the door and if these non-covalent bonds are distributed on one part of the doorframe, then the stability of the fit between a drug and a pocket will not be very good. Therefore, the distribution of all non-covalent bonds on each doorframe is also very important. Only with this distribution satisfying some threshold condition, we may find that increasing the value of F will promote the stability of the fit between a drug and a pocket.

We define the uniformity degree as:
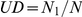
where
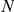
is the number of parts in the doorframe and
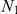
is the number of parts in the doorframe having non-covalent bonds. In practice, we require the threshold to be 1/N which equivalent to the requirement that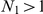, and the value of UD = 1 is the best.

Both the number and the uniformity degree of the non-covalent bonds can be computed using *Ligand Explorer*. As an example of the use of Ligand Explorer, we compute the number and distribution of non-covalent bonds for Oseltamivir, Zanamivir and Amantadine binding to pocket_2hu4 and pocket_1nyj, respectively.

In Table 3, A, B, C and D there are four domains that form the pockets. We find that

1. The binding forces for Oseltamivir, Zanamivir and Amantadine docked to pocket_2hu4 correspond to the energies of 95 kcal/mol, 75 kcal/mol and 75 kcal/mol, respectively. The UDs of Oseltamivir, Zanamivir and Amantadine in pocket_2hu4 are 1, 0.5 and 0.5, respectively.
2. The binding forces using Oseltamivir, Zanamivir and Amantadine to block pocket_1nyj corrspond to the energies 195 kcal/mol, 160 kcal/mol and 75 kcal/mol, respectively. The uniform degrees of Oseltamivir, Zanamivir and Amantadine on pocket_1nyj are 1.

**Table 3:** The amounts and the distribution of the non covalent bonds of Oseltamivir, Zanamivir and Amantadine on pocket_2hu4 and pocket_1nyj.

pocket	pocket_2hu4					pocket_1nyj				
Chain	A	B	C	D	total	A	B	C	D	total
Oseltamivir	4	4	9	2	19	8	2	18	11	39
Zanamivir	5	0	10	0	15	4	13	10	5	32
Amantadine	0	7	8	0	15	1	2	9	3	15

The binding force F under the condition
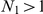
plays a very important role. Furthermore, F found for an arbitrary location always indicates a conditional binding force, and we still use the same symbol F, although this may create some confusion, so attention should be paid to the context. Nevertheless, the computation of F is not always carried out using the same rule. It should based on the molecular mechanism of interaction between a drug and a pocket. For example, if the pocket consists of two domains and the role of the drug is to bind to these two domains and prevent them from being separated from each other, then the value of F is the smaller of the two forces calculated for the two sides of the drug binding to the domains, respectively.

#### 4.3. The airtight degree between a drug and a pocket

According to the three features mentioned above, pocket_1nyj appears better than pocket_2hu4 for drugs Oseltamivir, Zanamivir and Amantadine. It is easy to understand that pocket_1nyj is better than pocket_2hu4 for Amantadine because pocket_1nyj is its benchmark pocket. However, pocket_1nyj appears to be better than pocket_2hu4 for Oseltamivir and Zanamivir. This is hard to understand since the benchmark pocket for Oseltamivir and Zanamivir is pocket_2hu4. This implies that we need more features to describe the drug's efficacy.

**Figure 2:**
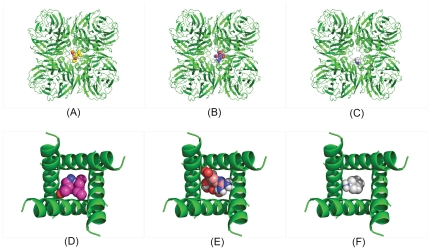
The drugs docking with pocket_2hu4 and pocket_1nyj. (A). Oseltamivir docking with pocket_2hu4. (B). Zanamivir docking with pocket_2hu4. (C). Amantadine docking with pocket_2hu4. (D). Oseltamivir docking with pocket_1nyj. (E). Zanamivir docking with pocket_1nyj. (F). Amantadine docking with pocket_1nyj.

From the diagrams of Oseltamivir, Zanamivir and Amantadine binding to pocket_2hu4 (Figure 2(A)–(C)) and pocket_1nyj (Figure 2(D)–(F)) we draw several pertinent observations. Based on these six figures, we find a common point that three drugs do not fill the two pockets as completely as possible. This may have implications for the drugs' limited efficacy. For example, the mismatch between Amantadine, Oseltamivir or Zanamivir and pocket_2hu4 is large enough that N-acetylneuraminic acid can be released and therefore it suggests that Amantadine, Oseltamivir or Zanamivir) is not effective in blocking pocket_2hu4. Similarly, the mismatch between Amantadine and pocket_1nyj is also large enough to pass ions through, and it also suggests that Amantadine (even Oseltamivir or Zanamivir) is ineffective in blocking pocket_1nyj if the ions produce large pressure. Therefore, it is necessary to introduce a measure of mismatch between a drug and a pocket it fills. We call it the airtight degree.

It is clear the airtight degree depends on the substance associated with the benchmark pocket. As mentioned above, the substance in pocket_2hu4 is N-acetylneuraminic acid while the substance in pocket_1nyj is a collection of ions but it was misunderstood as viral cytoplasm. We define the airtight degree as a 0–1 function: 1 if its substance cannot pass through it and 0 otherwise. This feature heavily depends on the knowledge of the substance filling the pocket.

#### 4.4. The inherent energy

Each channel must have an inherent force and its result is that the drugs cannot bind strongly enough to the protein surface. For example, the inherent energy of pocket_2hu4 is this energy to release N-acetylneuraminic acid. The inherent energy of pocket_1nyj is the exchange energy when the ions flow into the virus to eject viral cytoplasm. We have not been able to find relevant literature sources that discuss how to compute these inherent energies. However, we know that the inherent energy of pocket_2hu4 cannot be controlled by the concentration of ions while the inherent energy of pocket_1nyj can. This specificity will offset the weakness of Oseltamivir, Zanamivir and Amantadine binding to pocket_1nyj. Generally, the inherent energy of each channel has its specific mechanism and value, and therefore there is no unique way to compute it. Just this specificity offers an opportunity to design specifically effective drugs. Let F0 be the inherent energy of a channel. The value of F0 will make the drug ineffective if we ignore it. Therefore, we propose to introduce the inherent energy of pocket as a computable feature.

In summary, we have assembled a set of five computable features to describe the efficacy of a drug. It is clear that these five features can be translated into four decisions: D1 = max {0, -MFE}, D2 = max {0, F -F0}, D3 = max {0, UD-0.5} and D4 = max {0, ATD}. Finally, we can show that a drug or a drug & drug complex is effective if D1>0, D2>0, D3>0 and D4>0.

### 5 The universal algorithm to find drug & drug complexes

A universal program is used in this paper to search for an optimal drug & drug complex, which is inspired by the model of tight packing of balls into a box. We consider the pocket to represent the box and drug molecules to represent the balls. For clarity, we first describe the routine we use.

For a benchmark pocket, we first try to understand its molecular mechanism especially the type of the associated substance and then select the drugs from the DrugBank database to form the panel of drugs under study. In this panel, the known drugs that target this pocket should be included, the drugs which are as small as the associated substance also should be contained in the panel and drugs which are bigger than the known drugs aimed at this pocket should also be assembled in the panel for comparison proposed.

For a well-selected panel of drugs, we use the Autodock procedure to check each drug whether or not it can be packed into the given benchmark pocket. In practice, we do not add any prior information to the Autodock procedure, but let Autodock blindly dock a drug with a target protein using 5,000 iterations. We say the drug can dock with the benchmark pocket, if the minimum value of MFE within 5,000 iterations is negative and the position corresponding to the minimum value is contained in the benchmark pocket. We call it as the docked position.

The coordinates of a drug packed into a pocket are usually different from the original coordinates provided by DrugBank. If we regard the Autodock procedure as a mapping, then the drug with the original coordinates is the original object and the drug with the coordinates packed into a pocket is the image of the object. If we ignore the overlap among these images, then the envelope formed by the images of drugs is bigger than any single image of a drug. We say that a drug is a cover of the benchmark pocket if its image fills into the pocket fully enough so that the associated substance of the pocket cannot pass through the pocket. Generally, we say that a panel of drugs is a cover of the benchmark pocket if the envelope formed by the images of the drugs may fill the pocket fully enough so that the associated substance cannot pass through the pocket. In particular, a drug & drug complex is called a cover of the benchmark pocket, if the envelope formed by the images of the drug of drug &drug complex fill the pocket fully enough so that the associated substance cannot pass through the pocket and these images do not overlap with each other. In practice, some pockets may not have a cover even though the panel is enlarged to contain the entire DrugBank. Moreover, some benchmark pockets may not have the cover without an overlap even though they may have a cover. For convenience, we use C to denote the cover of the pocket induced by a panel of drugs.

If a benchmark pocket has an overlap cover C formed by N images (i.e., the C is formed by N images and these images may overlap), then we may further reduce it as the cover without overlapping by removing the redundant images from the cover C formed by the images of the panel. Using the combinations of traditional Chinese herbs as a metaphor, we can define the *king drug* and *minister drugs* in these combinations. We translate the concepts developed by the traditional Chinese medicine into the following typical circle searches:

#### Step 1

We select the largest image from the cover C as the “king” and denote it as K. We construct the subset c(K) of the cover by deleting all of those images which overlap with K from the cover.

If c(K) is empty, we output K alone and stop the search. Otherwise, we choose the largest image from c(K) as the “first minister” drug and denote it M1 and we construct c(K, M1) based on c(K) by deleting these images which overlap with M1 from c(K).

If c(K, M1) is empty, then we output K+M1 and stop the search. Otherwise, we select the largest image from c(K, M1) as the “second minister” and denote it by M2 and we continuously construct the subset c(K, M1, M2) based on c(K, M1) by deleting those images which overlap with M2 from c(K, M1).

If the c(K, M1, M2) is empty, then we output K+M1+M2 and stop the search. Otherwise, we choose the largest drug from c(K, M1, M2) as the “third minister” drug and denote it as M3 and construct c(K, M1, M2, M3) based on c(K, M1, M2) by deleting those images which overlap with M3 from c(K, M1, M2).

If c(K, M1, M2, M3) is empty, then output K+M1+M2+M3 and stop the search. Otherwise, we choose M4 from c(K, M1, M2, M3) and continue to construct c(K, M1, M2, M3, M4).

Continuing this procedure, the search stops at most at 5 circles because the sizes of the drugs in c(K) are less than half of the size of K, and the sizes of drugs in c(K, M1) are less than a half of the M1, the sizes of the drugs in c(K, M1, M2) are less than a half of M2, etc. Therefore, within at most 5 circles, the sizes of the rest drug will be less than the minimum size of all possible ligands.

Renewing the cover C with N images by moving the largest image K from C, we obtain the renewed cover C1 with N-1 images. We use C1 to replace the original cover C and repeat the above circle search. Inductively, we may renew the cover Ck based on the cover Ck-1. This renewal program terminates when the size of the largest image in Ck is less than the average size of the N images because in this case the drug &drug complex absolutely cannot cover the pocket.

#### Step 2

For all outputs corresponding to C, C1, C2,…,Cm, where, m is the maximal integer such that Cm is still a cover of the pocket, we check these m drug & drug complexes to determine each of them whether or not can prevent that associated substance pass through the pocket. This can be done either by visual inspection or by computation. We only output those entire drug & drug complexes which are the cover of the pocket.

The step 1 of the algorithm outputs only one drug & drug complex for each renewal exercise of the cover. As the renewing is finished, the number of outputs is at most N. Surely, the number may be less than N/2 if the panel has N drugs with balancing weights and we stop the renewing when the biggest drug in Ck is less than the average weight of C. For each circle search in step 1, the selection that is the largest in c(K), c(K, M1), c(K, M1, M2), etc. Just this cause, the outputs of our algorithm may not contain all covers without overlap. And therefore the final drug&drug complex may not be the optimum complex in the sense of computation. But it is suboptimal in some sense.

At the end, we analyze the total computational time for a given target protein and panel of drugs. The first cost is taken for N times to dock if the panel has N drugs. Autodock needs about 2 hours to confirm whether or not a drug can be packed into the pocket in our PC (2.83 GHz/3 G memory). The second cost is taken for search out the combinations of the *king drug* and *minister drugs*. The time is taken to do that depends on both the N and the weights of the drugs in the panel. But in practice the panel is not too large and the distribution of the weights is fair, which will save much time. For example, if we use our panel of 9 drugs, it only took a few minutes to finish this search. The third cost is to check the combinations whether or not satisfy D1>0, D2>0, D3>0, and D4>0. It takes at most 50 minutes if the F0 is known. Anyway, if the F0 is known, the time is no too long. However, in order to estimate a bound of F0, it takes 4–5 days if we use the molecular dynamic simulation to search the upper bound of F0.

## Results

To show how to use the universal program, we use pocket_2hu4 and pocket_1nyj as examples because their molecular mechanisms are well-known and the individually associated substance is also known. Below we list the following findings:

For pocket_1nyj, there is no drug & drug complex to fit into it.

For pocket_2hu4, since Vancomycin can not be packed into pocket_2hu4, then the cover is formed by 8 images of the rest drugs. For convenience, we do not distinguish the drug and its image. We arrange these 8 drugs orderly as Azithromycin, HEM, Heroin, Zanamivir, Oseltaminvir, Isosorbide, Aspirin and Amantadine according to the weights of the drugs shown in Table 1. Running the step 1, we then may find all drug& drug complex without overlap as below:

1. Azithromycin+Aspirin complex when Azithromycin is king.
2. HEM+Aspirin complex when HEM is the king.
3. Heroin+Amantadine complex when Heroin is the king.
4. There is no pair without overlapping when Zanamivir is king.
5. Oseltaminvir + Aspirin when Oseltaminvir is king.
6. We stop the circle searches for Isosorbide, Aspirin and Amantadine because their sizes are less than the average of all 8 drugs.

From this example, we may find that the algorithm to search the combinations of the king and ministers largely reduces the complexity. Comparably, if we enumerate all possible Drug&Drug combinations formed by these 8 drugs which may be packed into the pocket_2hu4, then the number of the possible 2-drug combinations is 28, the number of the possible 3-drug combinations is 56, the number of the possible 4-drug combinations is 70, and etc. It will take too much time to exclude all unsatisfied combinations. Among all possible 2-drug combinations, we may find that Azithromycin+Aspirin, HEM + Aspirin, Heroin + Amantadine, Oseltaminvir + Aspirin, Isosorbide + Aspirin, Aspirin +Amantadine and Amantadine+Isosorbide are all 2-drug combinations without overlap. This comparison shows that our algorithm does not loss the essential results, although the last three 2-drug combinations can not be output by the algorithm.

**Figure 3:**
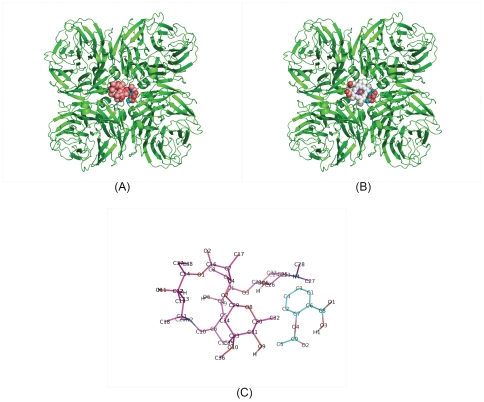
Two examples of drug & drug complexes. (A). The amplified picture shows that pocket_2hu4 was docked by Azichromycin & Aspirin complex. (B). The amplified picture shows that pocket_2hu4 was docked by HEM & Aspirin complex. (C). The picture to show that 2D topological diagram of Azichromycin & Aspirin complex.

We continue to analyze what combinations are the covers of pocket_2hu4 (step 2). Intuitively, Oseltaminvir+Aspirin cannot fill pocket_2hu4 fully enough to prevent N-acetylneuraminic acid (C_11_H_19_NO_9_) from being released. Therefore, Oseltaminvir+Aspirin cannot cover pocket_2hu4. With the same reason, Zanamivir alone and Heroin+Amantadine also cannot cover pocket_2hu4. For the rest two 2-drug complexes HEM+Aspirin and Azithromycin+Aspirin, HEM+Aspirin may fill pocket_2hu4 fully enough so that N-acetylneuraminic acid cannot pass through here (Figure 3(A)(B)). Moreover, the number of non covalent bonds between HEM+Aspirin and pocket_2hu4 is 45. Therefore, HEM+Aspirin should be a better drug&drug complex to be recommended. However, HEM is an unapproved drug and we can not use HEM & Aspirin to meet a clinical emergency situation. Comparably, Azithromycin+Aspirin is also a cover of pocket_2hu4, and the number of non covalent bonds between Azithromycin+Aspirin and pocket_2hu4 is 34. Therefore, we would like to recommend Azithromycin+Aspirin complex and we focus on to evaluation of the efficacy of the Azithromycin & Aspirin complex.

To show that the Azithromycin & Aspirin complex is predicted to be effective we need only to show that D2>0 and D3>0 because D1>0 and D4>0 are obvious.

Using Ligand Explorer, we easily compute the UD and F of the Azithromycin & Aspirin complex as follows: Azithromycin & Aspirin complex has 34 non-covalent bonds binding to pocket_2hu4 and they are distributed on all parts of the pocket_2hu4. Therefore, UD = 1 and then D3>0, which is obvious. Importantly, the combined force F is about 170 kcal/mol.

To ensure D2>0, we need to estimate the inherent F0 in pocket_2hu4. However, to measure F0 for pocket_2hu4 using experimental methods is presently impossible. Alternatively, we estimate the upper bound on F0 in pocket_2hu4 as 170 (kcal/mol). In other words, we predict that the Azithromycin & Aspirin complex is effective if F0 is less than 170 (kcal/mol).

The molecular dynamics simulation is regarded as the bridge between theory and experiment and between microscopic and macroscopic analyses. We may use the public software GROMACS to computationally observe how much energy may pull Azithromycin & Aspirin complex far from pocket_2hu4 in 2 angstrom. Typically, we let the moving speed of the spring be 0.001 nm/ps and the elasticity coefficients of the spring be 1,000,000 kJ mol^−1^ nm^−2^. Then we find that Azithromycin & Aspirin complex can not be squeezed out the pocket during 50,000 iterations (equals to 0.1 ns). We further check the distance between the geometrical center of Azithromycin & Aspirin complex and the geometrical center of pocket_2hu4, it is ranging from 0 to 0.8 angstrom, and the maximal energy during this 0.1 ns. Therefore, we may believe that distance is less than 0.5 angstrom is absolute reliable threshold to say that drug cannot be squeezed out, while it may be squeezed out if the distance is ranging to the interval from 0.5 to 1 angstrom. Next, we will use the minimal value of the energies when distance ranges into [0.5, 1.0] (unit: angstrom) as the critical value of energy so that drug cannot be squeezed out binding pocket.

When the elasticity coefficient of the spring is increased to 10,000,000 kJ mol^−1^ nm^−2^, then we may find that Zanamivir, Oseltamivir Azithromycin&Aspirin can be squeezed out pocket_2hu4 within 50,000 iterations. Then the critical energies of Zanamivir, Oseltamivir Azithromycin & Aspirin are 409 kJ/mol, 551 kJ/mol and 682 kJ/mol, respectively. Especially, 682 kJ/mol equals to 177.32 kcal/mol approximately. It is almost same as the estimated value through using the number of the non covalent bonds. It makes us sure the method used to estimate the critical energies is reliable. Further, we may infer that F0< min{409, 551} if Zanamivir and Oseltamivir are assumed as the effective drugs. It is certainly that Azithromycin &Aspirin docking with pocket_2hu4 satisfies D2>0 if F0<409.

We have produced a movie to show that Azithromycin & Aspirin complex cannot be separated from pocket_2hu4 using GROMACS with fixed pull energy 682 kJ/mol (Supporting Information S2). Note that the MD simulation only runs for 500,000 steps. It takes about 10 hours of CPU time to run it on our computer and it corresponds to what we observe in the actual situation of the Azithromycin & Aspirin complex docking with pocket_2hu4 for a 0.1 ns time span. In other words, if we wanted to observe the situation of the Azithromycin & Aspirin complex docking with pocket_2hu4 for one minute, this would require many years of computer simulations using a super computer.

To solve this very challenging computational problem, we collect a sequence of distances from the geometrical center of the pocket to the geometrical center of the drug over 50,000 steps. We regard this sequence as the observed data of a stochastic process. Using the signal processing approach, we can prove that Azithromycin & Aspirin complex docks with pocket_2hu4 tightly for ever. The detail proof is omitted. Anyway, simulation results support that Azithromycin & Aspirin complex should be considered to do the clinical experiments.

## Discussion

In spite of the fact that state-of-the-art computational prediction methods offer a high level of confidence, clinical validation provides the final decision. It will be very reassuring if the efficacies of these drug & drug complexes studied here are validated clinically because the Azithromycin & Aspirin complex may be readily used to treat all subtypes in group N1.

Fortunately, an easy and safe way can be used to provide clinical validation. For example, using the dosage: 1 Azithromycin dispersible tablet (0.1 g/tablet) and 2 aspirin enteric-coated tablet (0.1 g/tablet), one of the authors, J Ruan experimented on himself. After two hours following the drug administration sneezing and sniffling stopped. The remaining symptoms disappeared after 12 hours following the second dosage. Of course, large scale clinical tests are needed to confirm this anecdotal observation.

For pocket_1nyj, there is no drug & drug complex to fit it. However, Oseltamivir, Zanamivir or Amantadine may be conditionally effective when they bind to pocket_1nyj. In fact, we have also simulated the situation for Oseltamivir binding to pocket_1nyj within 2 ns of simulation time. When the moving speed of the spring is 0.001 nm/ps and the elasticity coefficient of spring is 1,000 kJ mol^−1^ nm^−2^, Oseltamivir is separated from the pocket_1nyj. In this case, F0 is about 58.4 kJ/mol. Therefore, if the concentration of ions could be regulated down so that F0 is much less than 58.4 kJ/mol, then D2>0. Therefore, it could then become effective. We may find the following implications of this conclusion.

It is possible to deduce why Oseltamivir is effective to treat H1N1 in humans but not effective in avian species [20]. In fact, Oseltamivir cannot inhibit the release of N-acetylneuraminic acid and therefore it is useless to block pocket_2hu4. Comparably, Oseltamivir can dock with pocket_1nyj even though it is not too airtight to block the ions' entry inside the bilayer, but it can prevent the viral cytoplasm from flowing out of the lipid envelope when the exchange energy F0 is not larger than the binding force F of Oseltamivir. The specific characteristic of the inherent energy F0 in pocket_1nyj can be controlled by changing the concentration of ions. Both humans and swine can drink large quantities of water in a short period of time and therefore the concentration of ions is reduced which results in reducing the value of F0 for pocket_1nyj. With this insight, we can understand why Oseltamivir is not efficacious for avian species [20] since birds are unable to drink much water in a short period of time.

The Azithromycin & Aspirin complex may be chemically bound using covalent bonds to become a new drug. In this way, the dosage of the new drug could be reduced which may avoid some side effects due to the administration of Azithromycin or Aspirin individually. For example, the Azithromycin & Aspirin complex can be linked according to the distance matrix or the picture shown in Figure 3(C). Of course, when it becomes a new drug it will require more time and a high cost to determine its pharmacokinetics, pharmacodynamics, side-effects and efficacy.

In summary, the five computable features proposed in this paper are used to assess the efficacy of a drug or the referential features to design novel drugs and drug combinations. Among these five features, the airtight degree and the inherent force are difficult to compute because they depend on the substance present in the benchmark pocket. Unfortunately, we have no good methods to estimate the inherent force at present which limits the application of the drug & drug complex prediction methods until we can accurately estimate the inherent forces. The four decisions are the mathematical expressions of these five features. The key is to check that D2>0. The condition D2>0 will lead to a more predictable situation for the design of drugs.

In closing we briefly summarize the key insights arrived at in this paper. Drug resistance is most often used to explain the lack of efficacy of approved drugs and is usually linked to the presence of mutations. However, in our view, this may be incomplete or incorrect and could become a limiting factor in drug design. In fact, the benchmark pockets of all approved drugs are found to be highly conserved but the drug resistance still occurs. It is possible that the real cause for the approved drugs to lose efficacy is because some computable features have been ignored. There are multiple causes of drug resistance and we need more computable features to measure them. Urgently, we wish some readers may develop an experimental method to measure the F0.

## Supporting Information

Supporting Information S1 The document is presented for details of assemble the panel of drugs and explanation of drugs docking in the same pocket.
(DOC)

Supporting Information S2 The movie to show that Azithromycin & Aspirin complex cannot be separated from pocket_2hu4 when the elasticity coefficient of the spring is 1,000,000 kJ mol nm^−2^
(GIF)
