# Supplementary material for: Computable Features Required to Evaluate the Efficacy of Drugs and a Universal Algorithm to Find Optimally Effective Drug in a Drug Complex
Source: PLoS One. 2012 Mar 23;7(3):e33709. doi: 10.1371/journal.pone.0033709 (PMC3311648; doi:10.1371/journal.pone.0033709)
Supplement: Supporting Information S1 — The document is presented for details of assemble the panel of drugs and explanation of drugs docking in the same pocket. (DOC) [file pone.0033709.s001.doc]

Supplement for

Computable features required to evaluate the efficacy of drugs and a universal algorithm to find optimally effective drug in a drug complex

Kui Wang, Wei Cui, Gang Hu, Jianzhao Gao, Zhonghua Wu, Xingye Qiu, Jishou Ruan, Yi Feng, Zhi Qi, Yiming Shaoand J.A. Tuszynski^4,5^

College of Mathematical Sciences and LPMC, Nankai University, Tianjin, PRC

National Center for AIDS/STD Control and Prevention, Chinese Center for Disease Control and Prevention, Beijing, PRC

National Key Laboratory for Pharmaceutical chemistry and biology at Nankai University, Tianjin, PRC

^4^ *Department of Physics, University of Alberta, Edmonton, AB, Canada*

^5^*Department of Oncology, University of Alberta, Edmonton, AB, Canada*

*Corresponding author: [jsruan@nankai.edu.cn](mailto:jsruan@nankai.edu.cn)

1. **How to assemble the panel of drugs**

The panel of drugs is consisted of 9 drugs. Amantadine, Oseltamivir and Zanamivir are certainly selected because we know their benchmark pockets and their background in detail. However, the choice of the 6 non-flu drugs is really arbitrary. There is a long history related to this choice. While we are interested in drug design, we are not experts in pharmacology. On a personal note, one of the authors, Dr. JRuan, is a patient with heart trouble and he takes Aspirin and Isosorbine and some other drugs every day. Therefore, he knows from personal experience that these drugs are safe. We found they have smaller molecular weights and we checked they are included in DrugBank so we could determine the coordinates of the drugs. Azithromycin and Vancomycin are two popular antiphlogistic drugs, but we were not familiar with them before we studied influenza viruses. Heroin and HEM are included in the panel only for comparison purposed. Since we wanted to search for drugs having acetyl groups, since Aspirin has acetyl, and found out that Heroin and Azithromycin are larger than aspirin and they have acetyls and methyl, they were therefore included in the study. Moreover, they are not the influenza drugs. Heroin is a natural opioid anelgesic and Azithromycin is a popular antiphlogistic drug having low side-effects but long half life. Therefore, they were chosen for the panel. At first, we wanted to use the 7 drugs: Aspirin, Isosorbine, Herion, Amantadine, Zanamivir, Oseltamivir and Azichromcyin to form the panel to evaluate the efficacies of Amantadine, Zanamivir and Oseltamivir and we wanted the number of drugs in the panel to be odd. When we computed the sizes of these seven drugs we found the distribution of the sizes was too narrow. Since the size of Heroin is not significantly larger than the maximal size of Amantadine, Zanamivir and Oseltamivir, then only Azichromcyin’s size is greater than the maximal size of Amantadine, Zanamivir and Oseltamivir. To make the panel better balanced in size, we looked for two drugs with sizes bigger than the maximal size of Amantadine, Zanamivir and Oseltamivir. We found out that Vancomycin is larger and is an approved drug, thus we chose it for the panel. HEM is a natural product contained in DrugBank but is not an approved drug. Also, it is a larger ligand. Therefore, we included it in the panel. Then the final panel of 9 drugs is fair in size and contains a balance between positive samples and negative samples. Luckily, according to the hint of the anonymous reviewers, we further search out the experimental target proteins of the 9 drugs from DrugBank, which may obviously tell us that 6 drugs we selected randomly are really non influenza viral drugs. And we summarize the basic knowledge of the 9 drugs in the Table S1.

Table S1. The weight and the target protein of the drugs in the panel

| drug | weight | Target protein | groups |
| --- | --- | --- | --- |
| Vancomycin | 1449.254 | 1pnv | approved |
| Azithromycin | 748.9845 | **50S ribosomal protein L4** | approved |
| HEM | 618.46 | 1bep | experimental |
| Heroin | 369.411 | **Mu-type opioid receptor** | illicit, experimental |
| Zanamivir | 332.3098 | NA (**Neuraminidase)** | approved |
| Ossltamivir | 312.4045 | NA (**Neuraminidase)** | approved |
| Isosorbide | 191.1388 | enzyme guanylate cyclase | approved |
| Aspirin | 180.1574 | COX-1/ COX-2 | approved |
| Amantadine | 151.2487 | Proton channel protein M2 | approved |

1. **The detail to validate why all drugs may be predicted to be docked with same pocket**

Many years ago, we only process one ligand and one pocket at a time. When we studied the influenza viral drugs: Amantadine, Zanamivir and Oseltamivir, we downloaded 1nyj and 2hu4 from PDB since the target protein for Amantadine is 1nyj and the target protein for Zanamivir and Oseltamivir is 2hu4. Then we found out that Amantadine, Zanamivir and Oseltamivir can be docked into pocket_2hu4 and pocket_1nyj the same way but did not utilize it. When we compared the efficacies of Amantadine, Zanamivir and Oseltamivir with other non influenza viral drugs, we found that all drugs selected can be docked in the same neighborhood of a pocket. As the number of the drugs increases, this phenomena is unchanged. We were puzzled by the phenomena. On one occasion, we suspected either we had misused the AutoDock or AutoDcok has some flaw because different drugs may be predicted to dock with the same place.

We first exclude that we had operated AutoDock wrongly because we had validated that Oseltamivir and Zanamivir can find their benchmark pocket on their target protein NA, that Amantadine can also find its benchmark pocket on its target protein M2, and that Fosamprenavir, Indinavir, Nelfinavir, Darunavir, Tipranavir and Amprenavir can also find their benchmark pocket on their target protein HIV-1 protease.

We also exclude that AutoDcok has flaw after we validated this result on a large panel of proteins and a large panel of ligands. In fact, selecting 1rd8, 2hu4, 1nyj, 3cm8, 3hw3, 1g6l, 2jle, 2gv9, 3gbn, 3gbm, 3fku, 3sdy, 3ztn and 3ztj as the target proteins, and choosing Amantadine, Aspirin, Azithromycin, HEM, Heroin, Isosorbide, Oseltamivir, Zanamivir and Vancomycin as the panel of drugs, then all of these ligands are predicted to be docked with the same pocket on each of above target proteins if these ligands can be packed into this pocket, while all of those ligands will arrive at the minimal value of minimal free energy at a neighborhood of the pocket if those ligands can not be packed into this pocket. Moreover, when the panel of ligands is enlarged to 34 ligands (as shown below), these 34 drugs are also predicted to be docked with the same pocket uniformly.


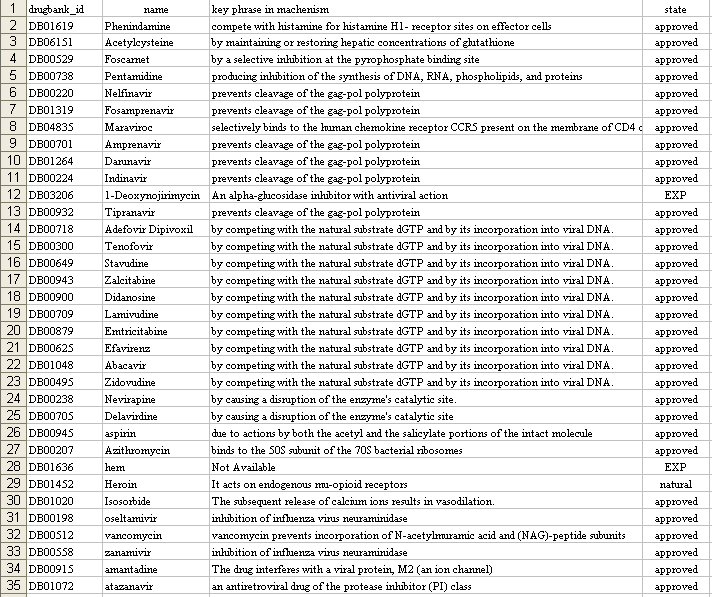


Furthermore, for the proteins formed by subunits, if we just use a subunit as the target protein, then all ligands also are predicted to be docked with the same pocket on the subunit. Of course, this pocket on a subunit is not same as that pocket on entire protein. For example, using a subunit of 3hw3 and entire 3hw3, we have two pockets shown as below:

| 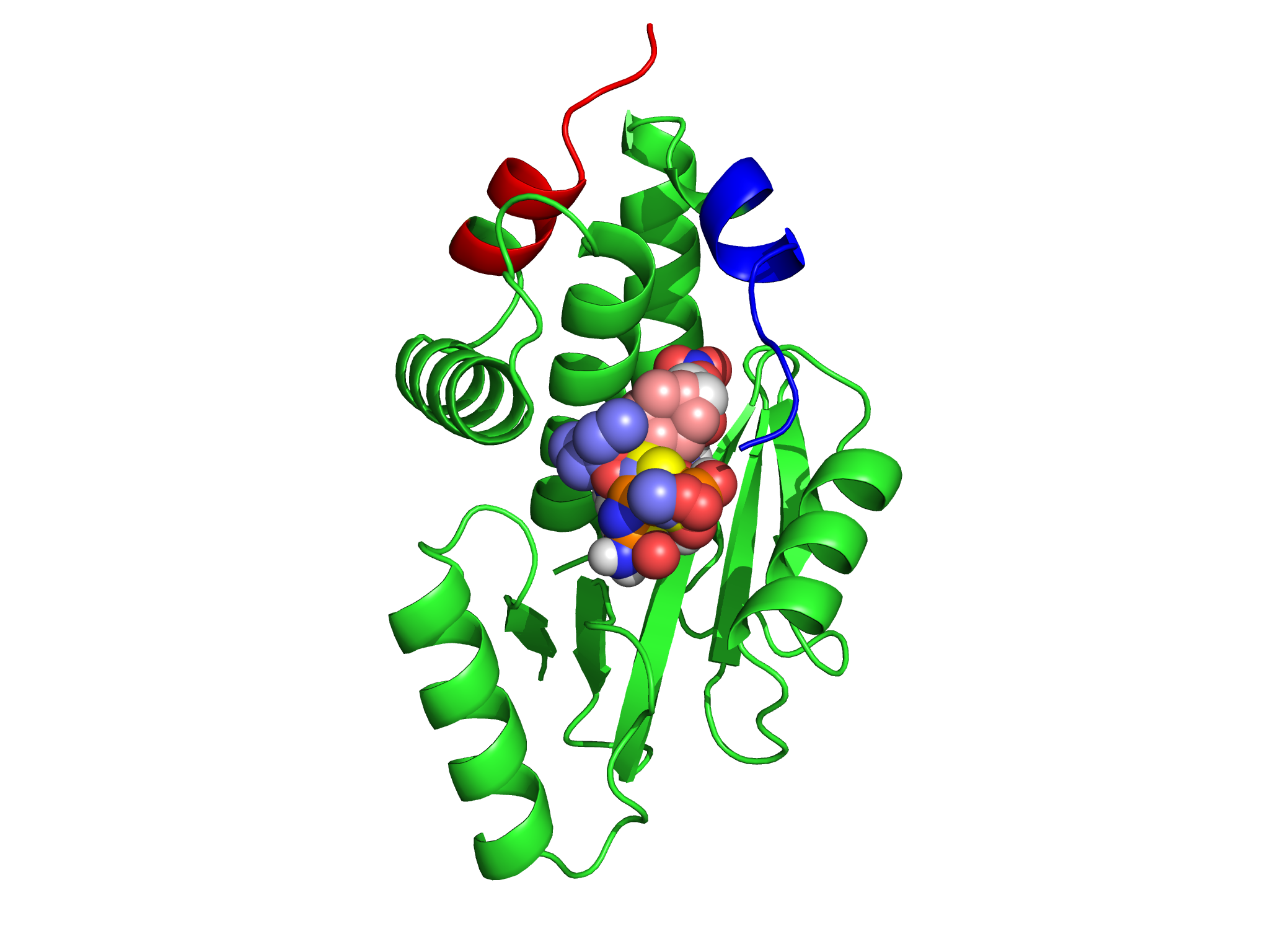 | 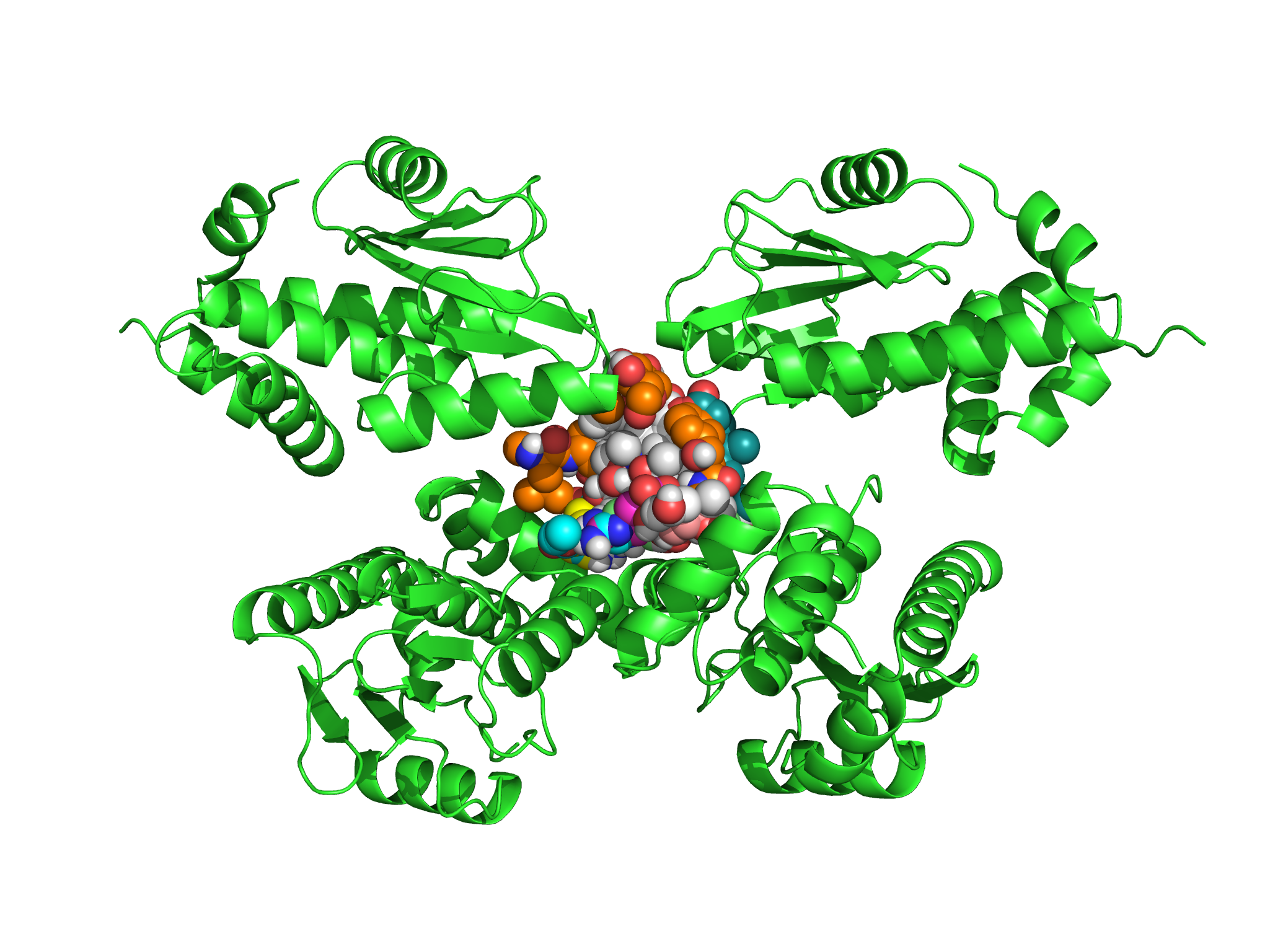 |
| --- | --- |
| Figure S1. Drugs are packed into the position of the metallic ions if we use a subunit of 3hw3 as the target protein. | Figure S2. Drugs are packed into the void surrounded by four subunits if we use 3hw3 as the target protein. |

Another example, we use the complex proteins formed by HA and antibody (i.e., 3gbn, 3gbm, 3ztj, 3ztn, 3fku and 3sdy) as the target proteins, then the pockets are shown as below:

| 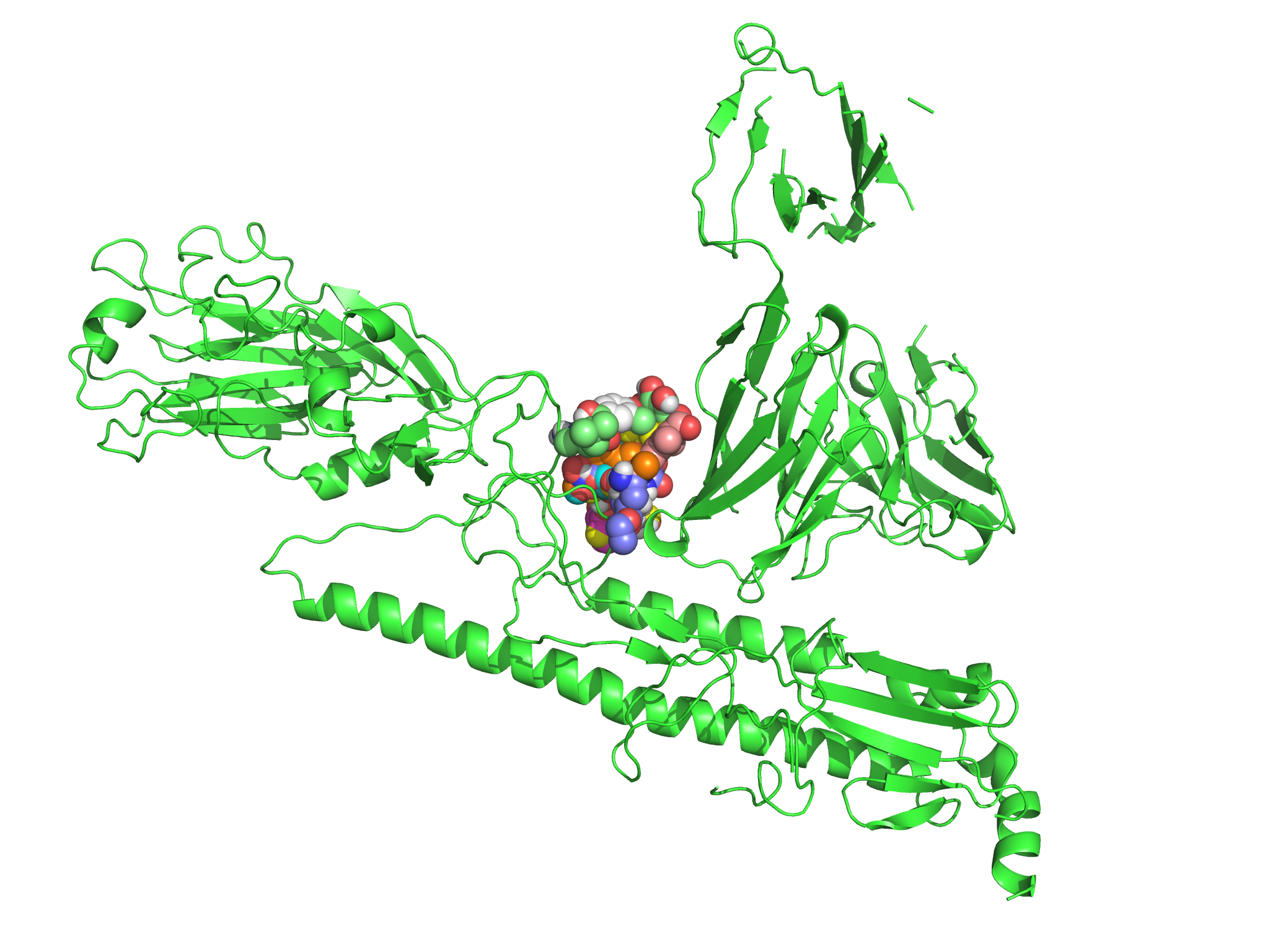 | 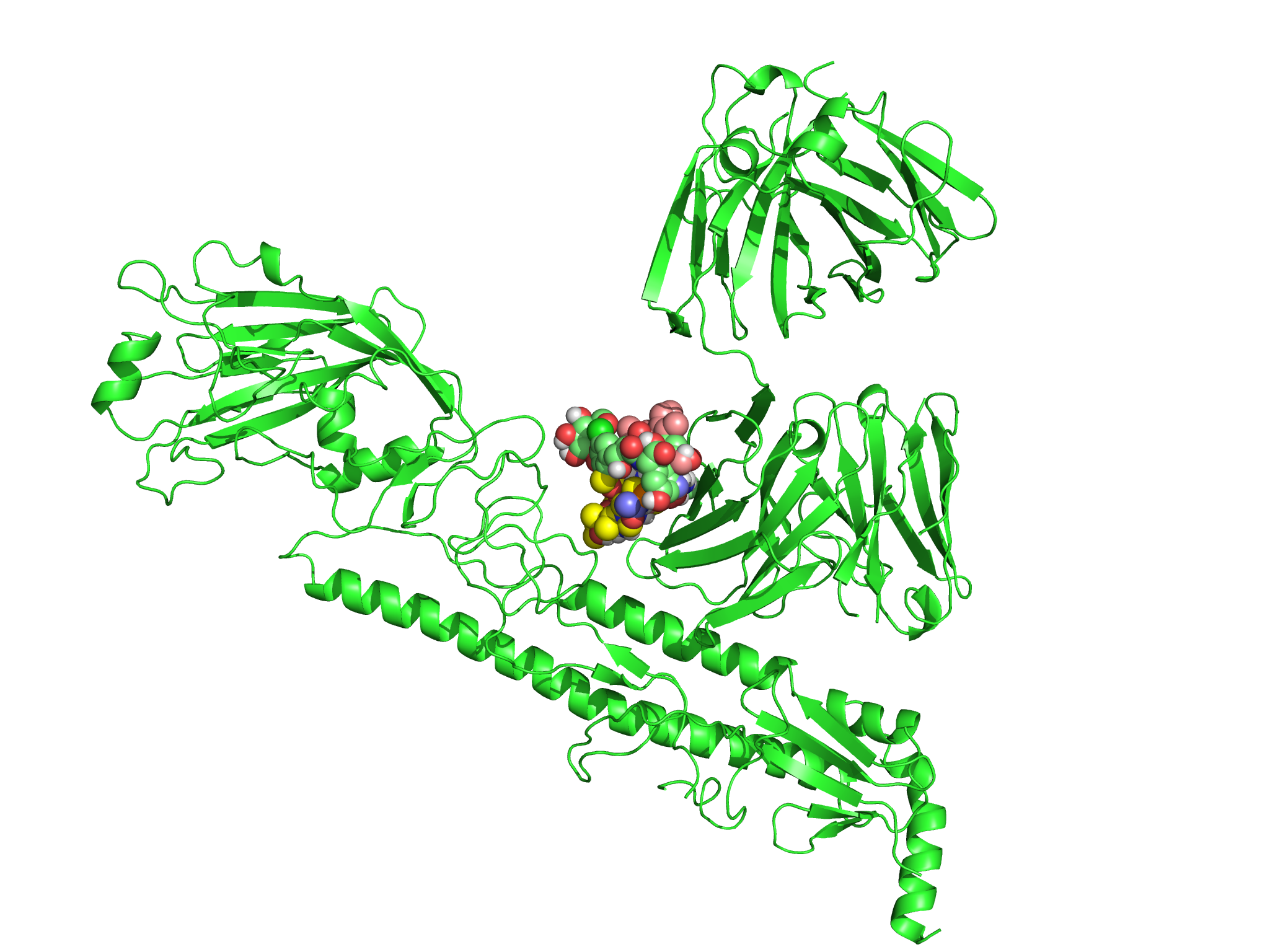 |
| --- | --- |
| Figure S3. 9 drugs docking with 3gbn | Figure S4. 9 drugs docking with 3gbm |
| 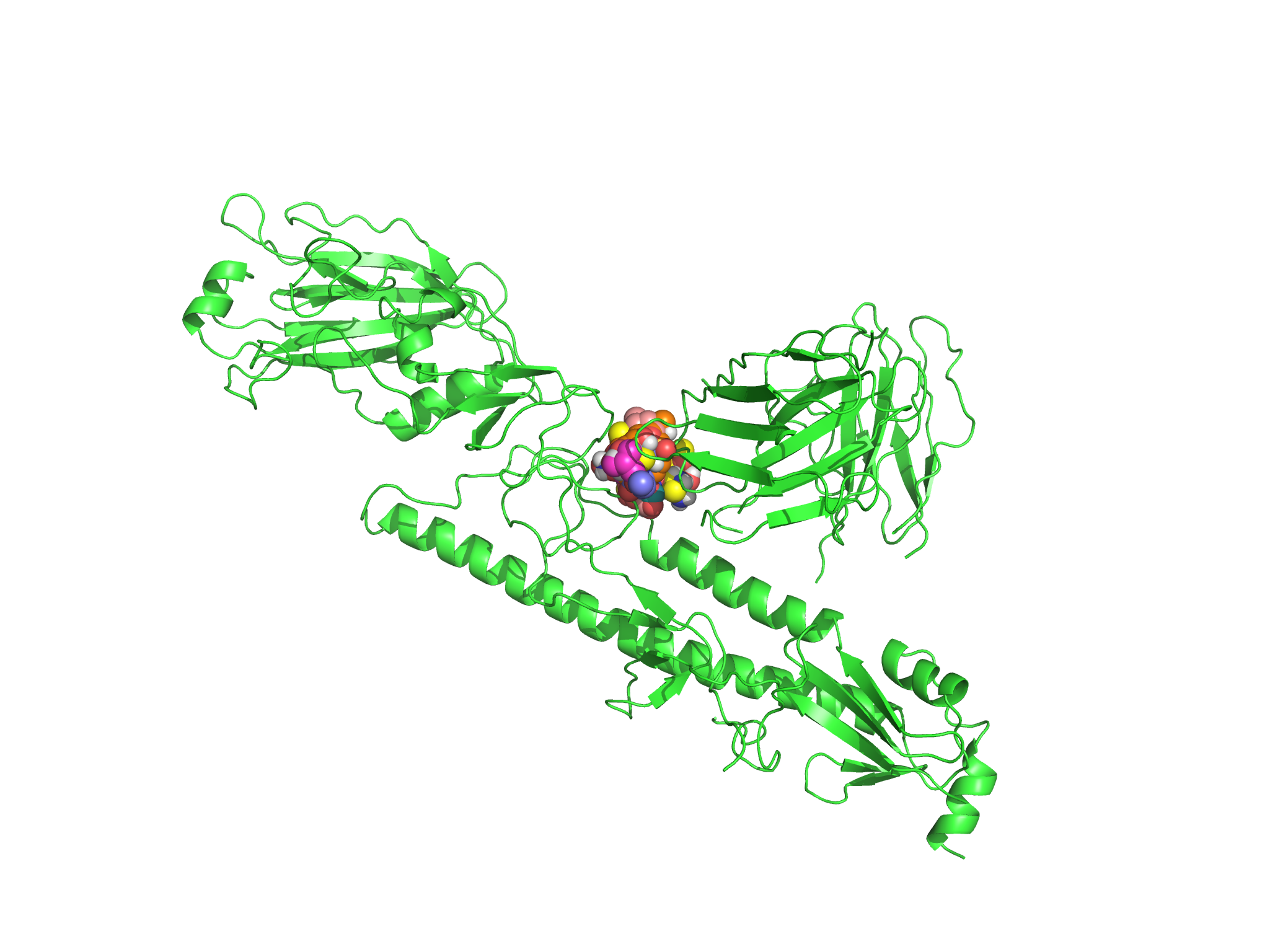 | 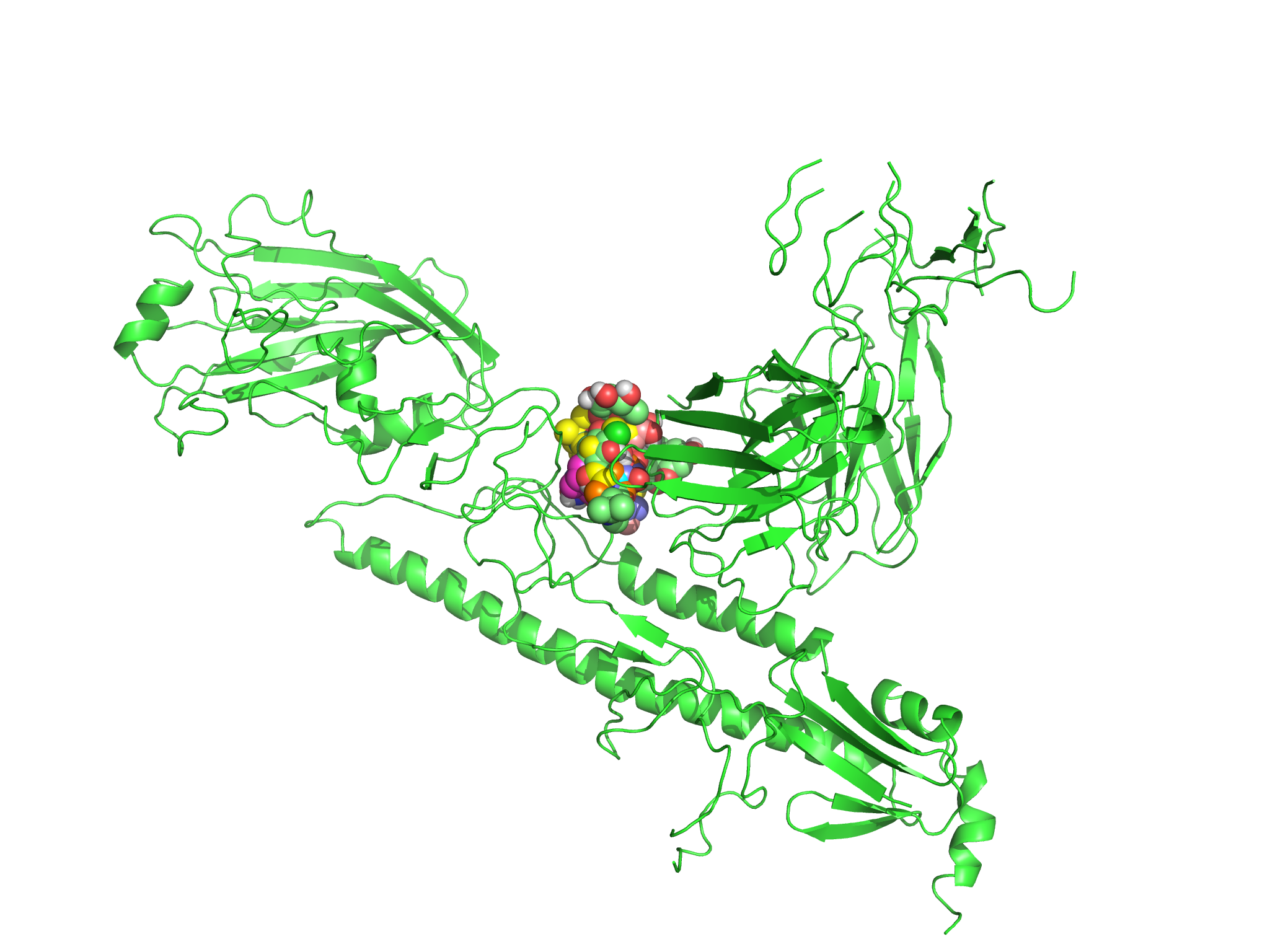 |
| Figure S5. 8 drugs docking with 3tzj | Figure S6. 9 drugs docking with 3tzj |
| 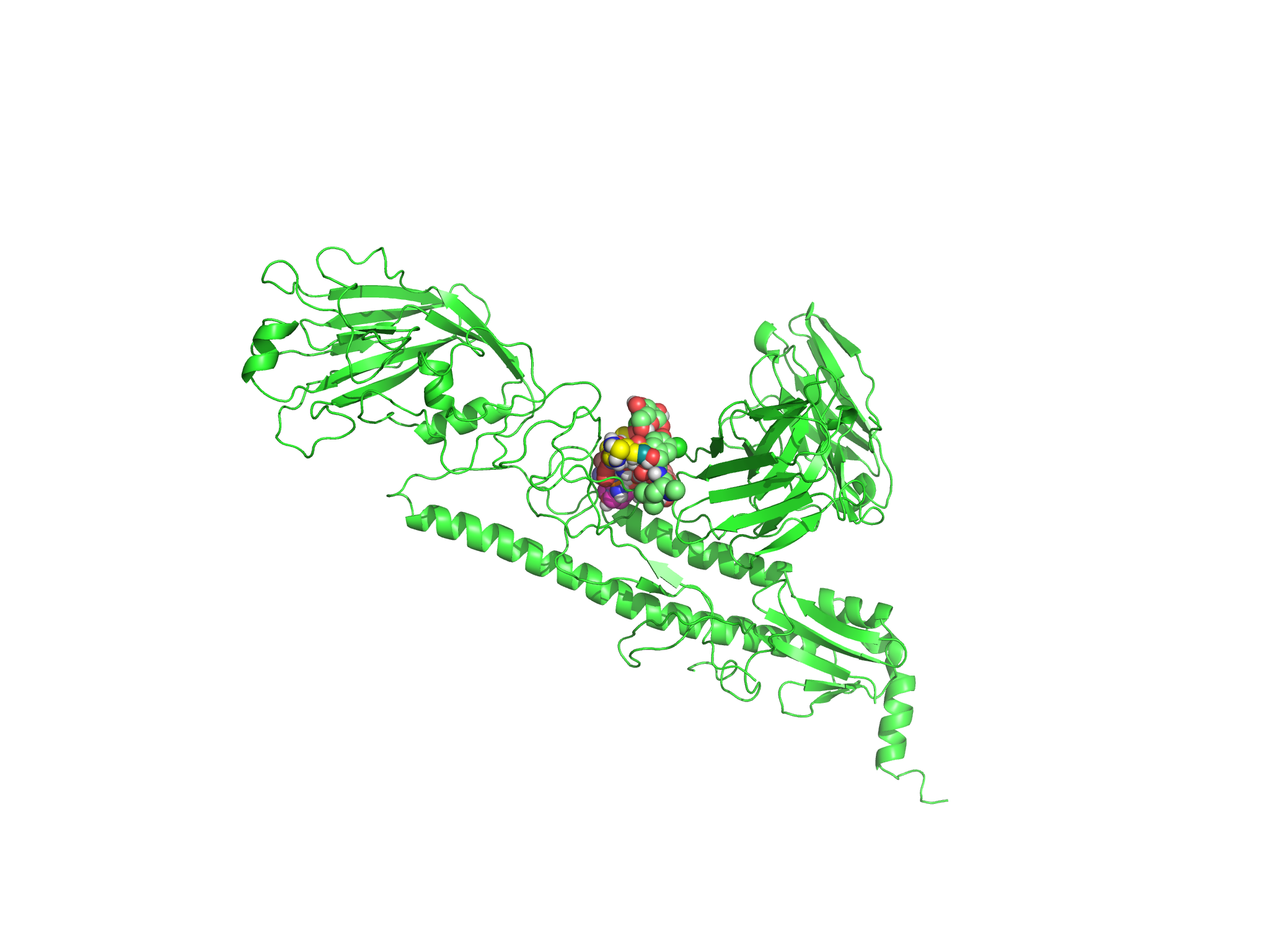 | 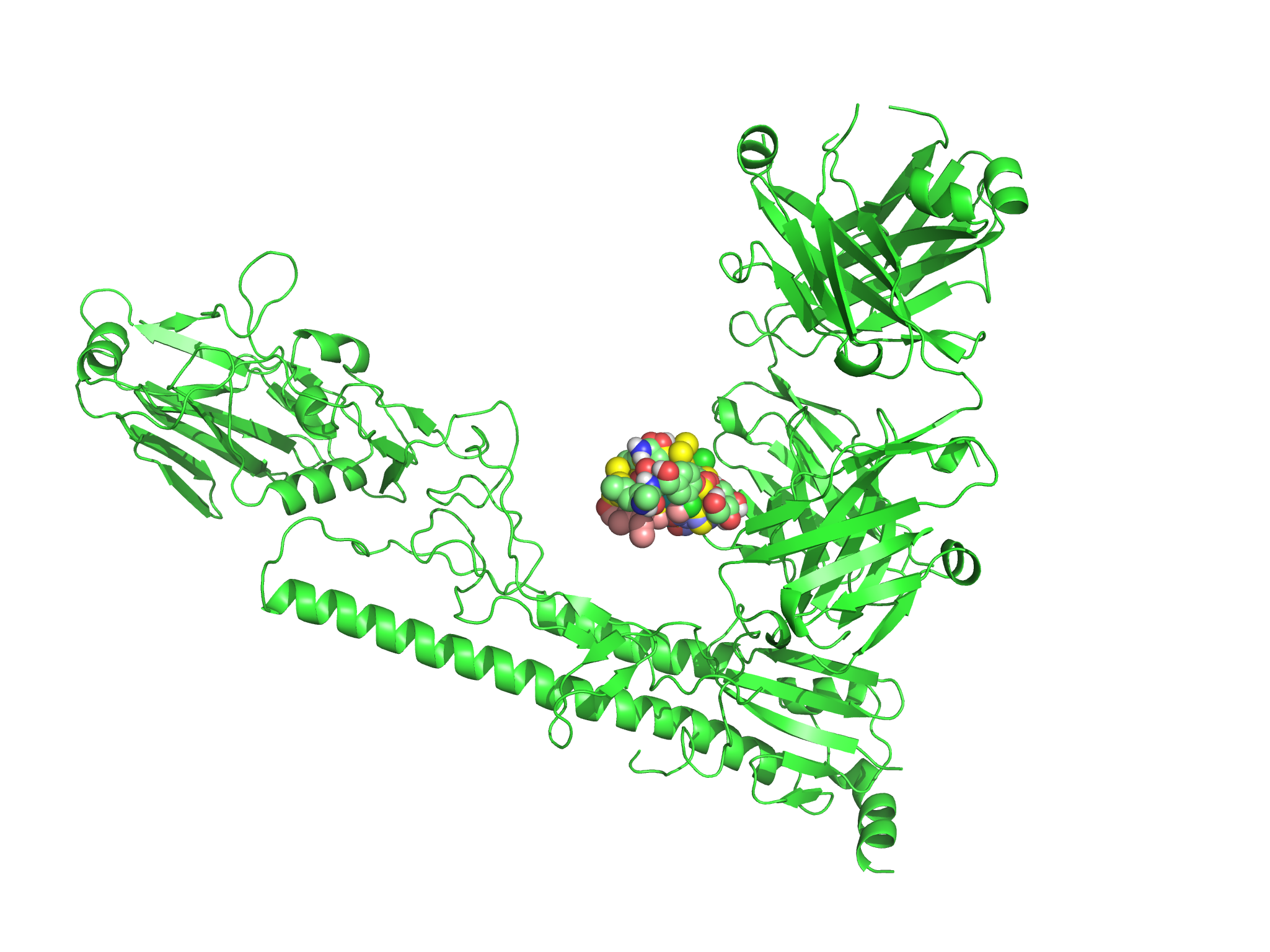 |
| Figure S7. 9 drugs docking with 3fku | Figure S8. 9 drugs docking with 3sdy |

However, if we use CR6261, F10, CR8020 and FI6 as the target proteins, then the pocket on each antibody is quite different from above fork. We show the pockets as below:

| 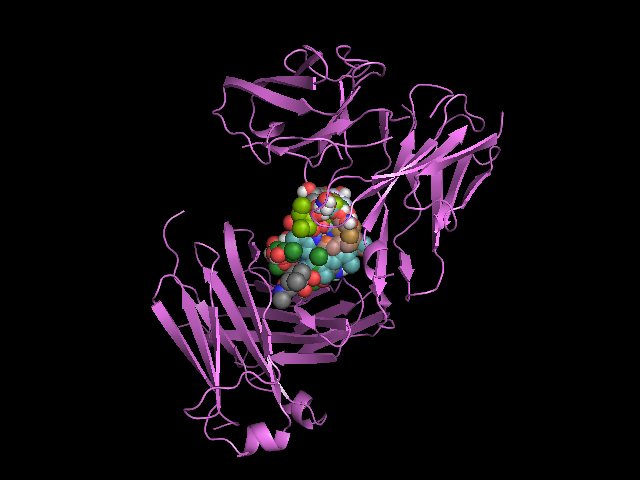 | 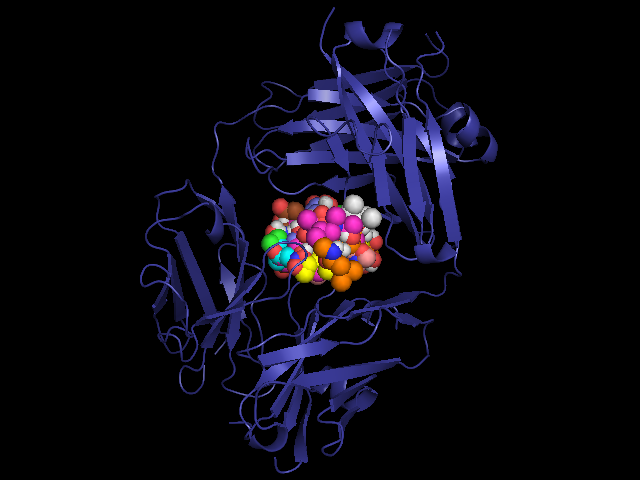 |
| --- | --- |
| Figure S9. pocket on CR6261 | Figure S10. pocket on CR8020 |
| 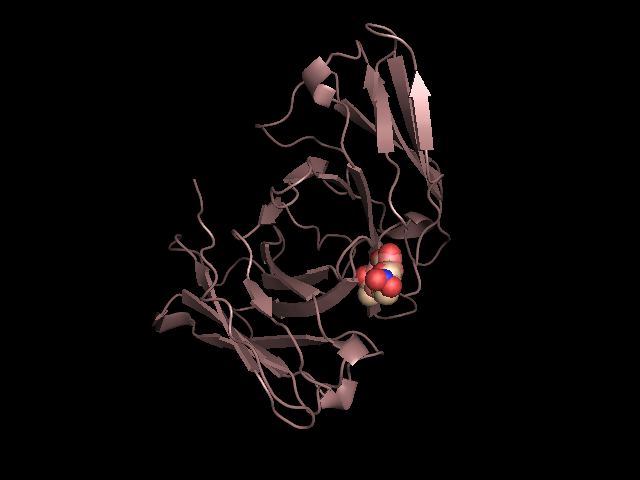 | 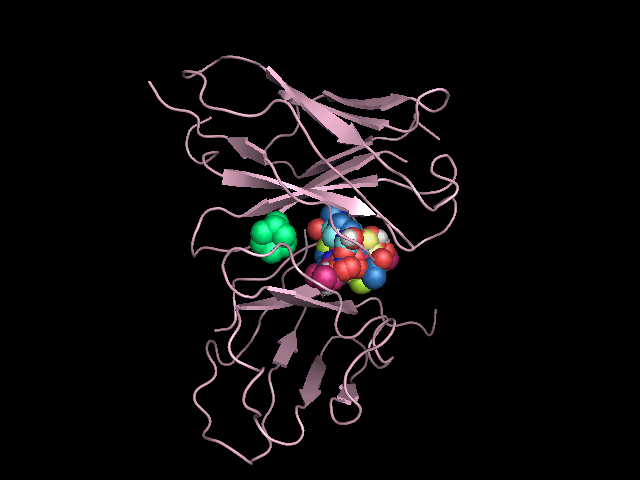 |
| Figure S11. pocket on F10 | Figure S12. pocket on FI6 |

If we use H1 HA, H5 HA, H3 HA and H7 HA as the target proteins, the pocket on each class are almost same shown as below:

| 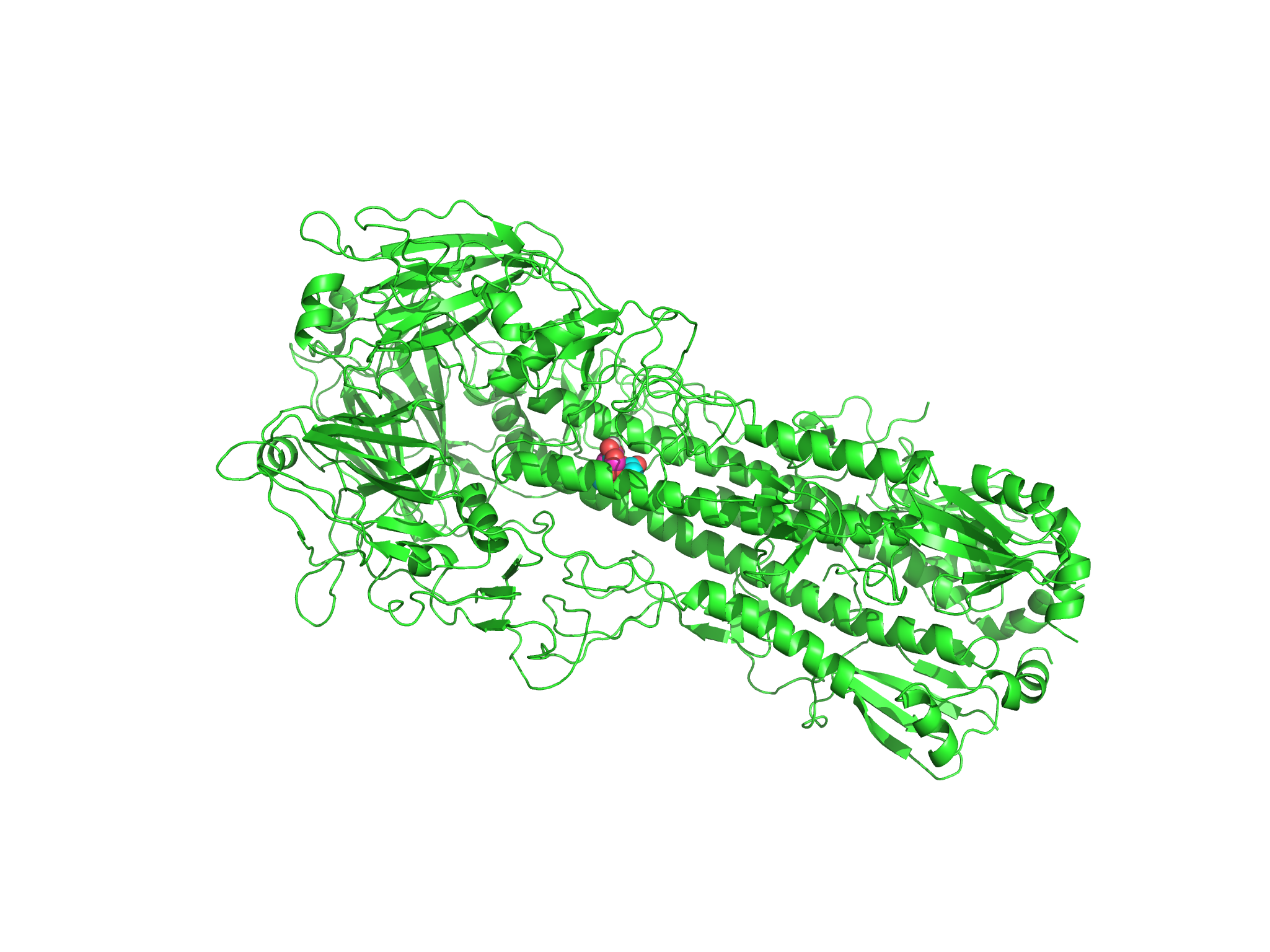 | 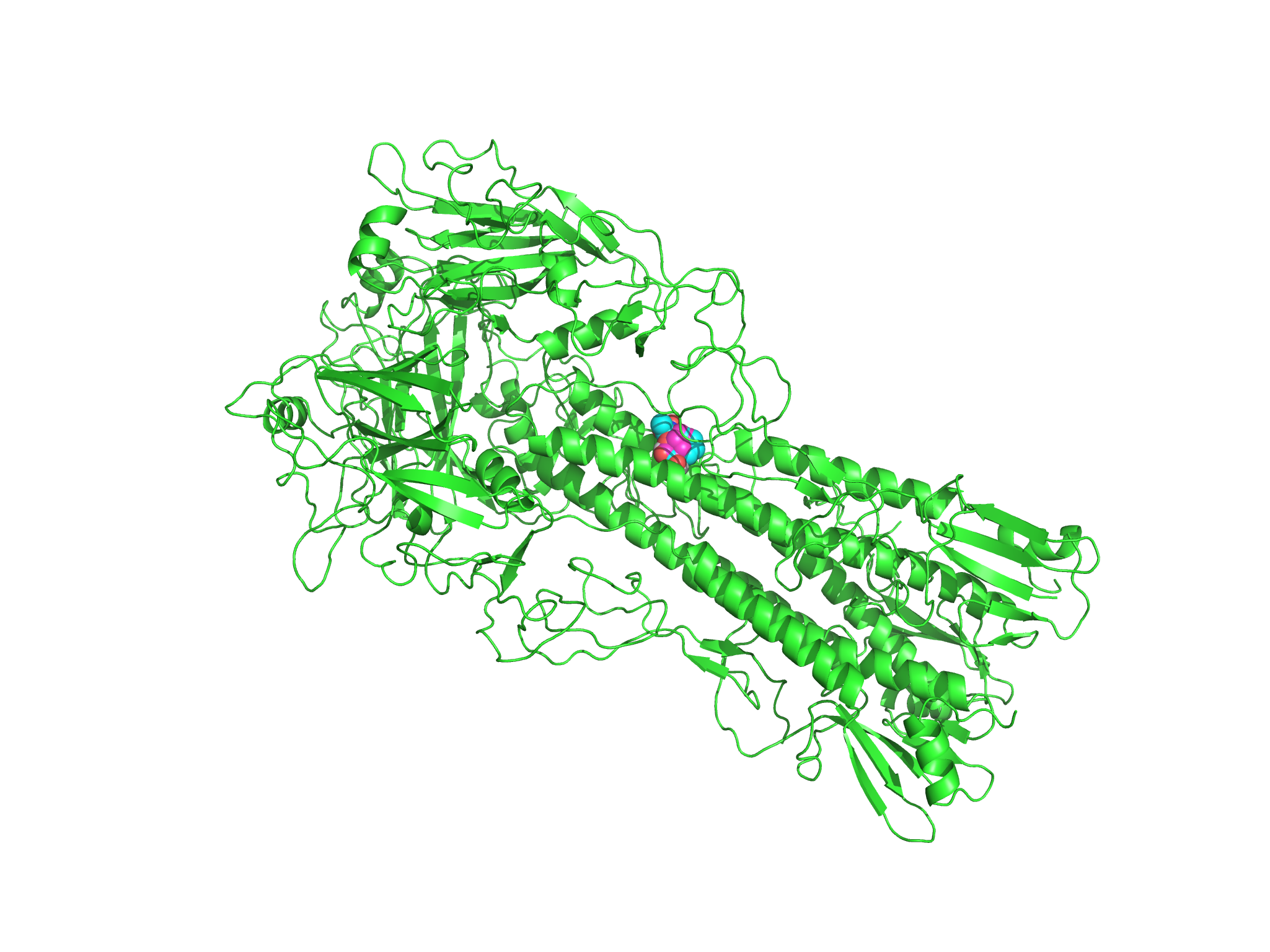 |
| --- | --- |
| Figure S13. The pocket on 1qml | Figure S14. The pocket on 2ibx |
| 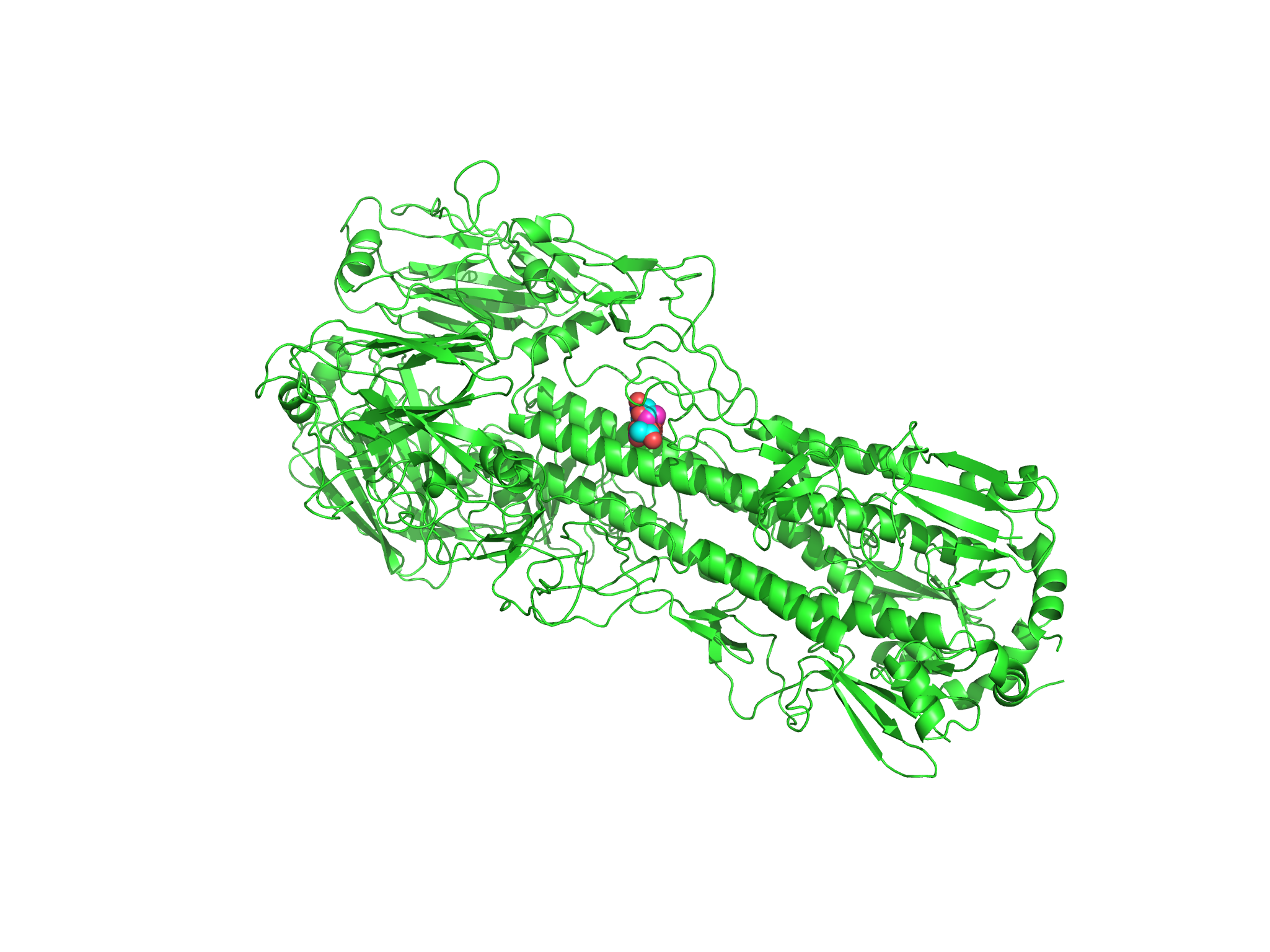 | 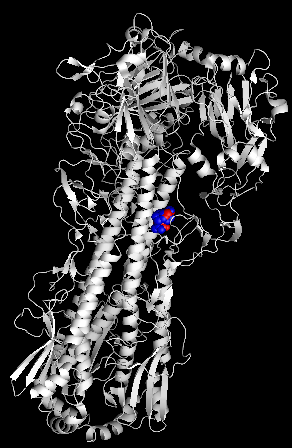 |
| Figure S15. The pocket on 3m5g | Figure S16. The pocket on 1rd8 |

After above validation, not only we confidently trust that AutoDock is a reliable tool to find the benchmark pocket of drugs on the given target protein, but also we believe that AutoDock must contain a good preprocessing subprogram so that AutoDock always may escape from the trap of the locally minimal value. Encouraged by this advantage, we have the idea to utilize this advantage sufficiently. In fact, if we input the 3D coordinates of a drug and the 3D coordinates of a protein, then AutoDock will outputs a value of the minimal free energy (MFE) and a predicted coordinates of the drug. Also, if we input a panel of drugs with the 3D coordinates and the 3D coordinates of a protein, then AutoDock will output a series of values of MFE and the predicted coordinates of the drugs. Therefore, if we show out all of these drugs with negative MFE using PyMOL according to the predicted coordinates at same time, then these drugs will be clustered in a void or a groove. And then we say this void/groove on the given target protein is a benchmark pocket of these drugs.

Nevertheless, AutoDock still has a minor flaw. In practice, the predicted docking fashion may not be perfectly same as the real fashion observed using x-ray. For example, the complex structure of Indinavir docking with 2bpx (one type of HVI-1 protease) obtained through x-ray is not perfectly same as the complex structure of Indinavir docking with 2bpx predicted using Autodock, through both they are packed into the same benchmark pocket.

| 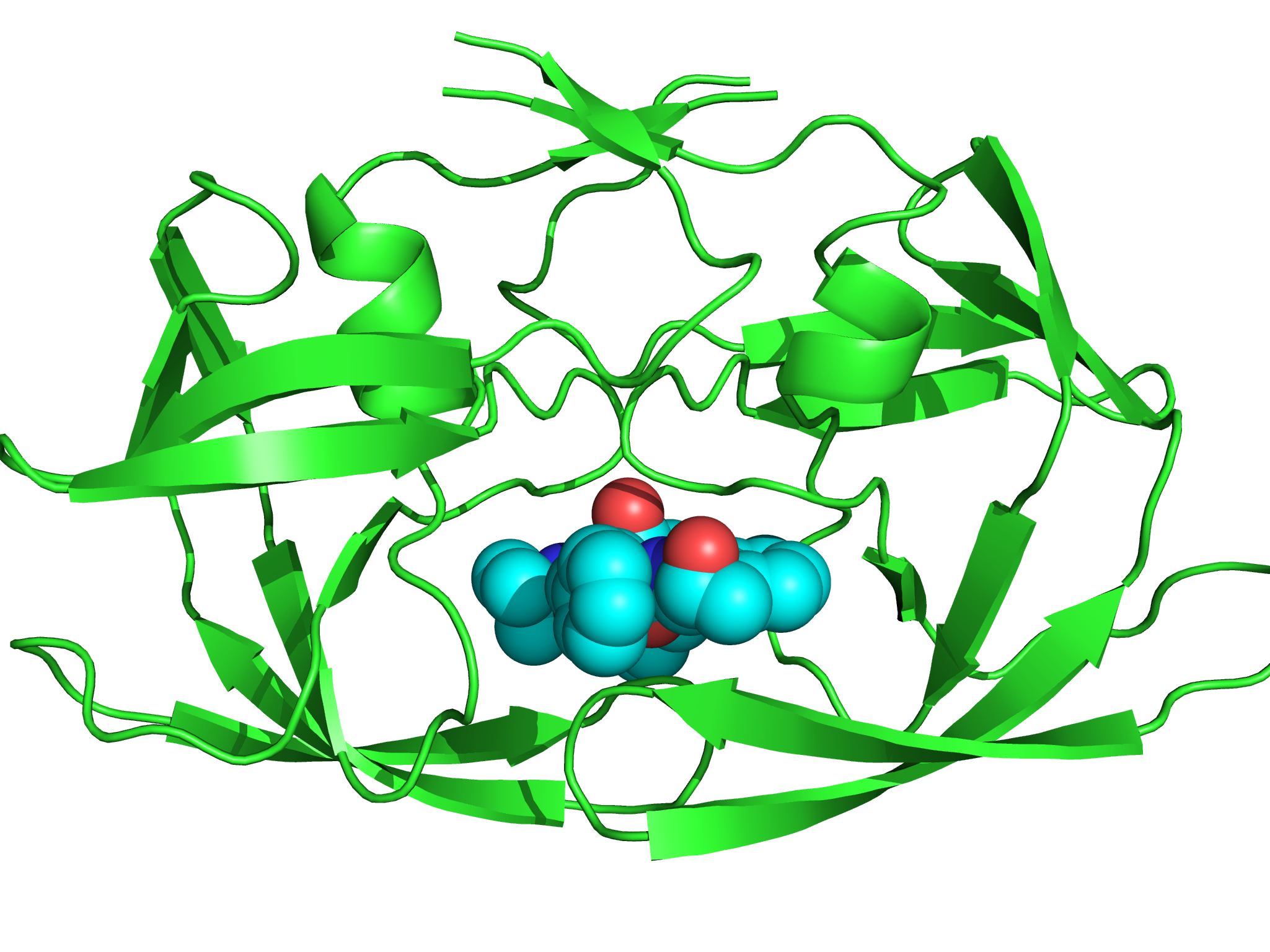 | 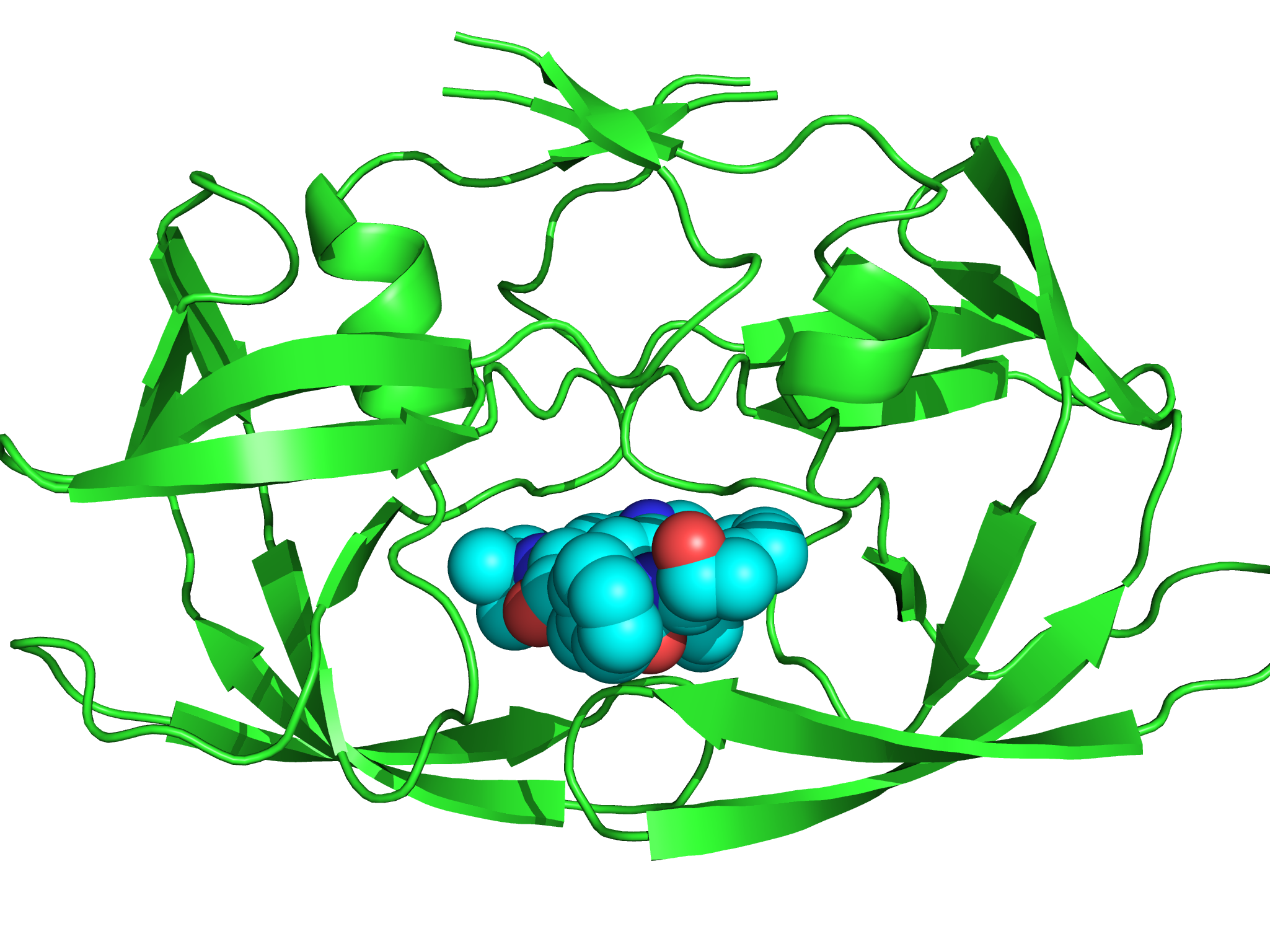 |
| --- | --- |
| Figure S17. The experimental effect of Indinavir binding to the pocket on 2bpx | Figure S18. The docking effect of Indinavir binding to 2bpx using AutoDock |

Consequently, we should keep in mind that predicted efficacy of a dug may not be perfect same as the real case. It is just a good referential answer.
