# Supplementary figures and images for: Computable Features Required to Evaluate the Efficacy of Drugs and a Universal Algorithm to Find Optimally Effective Drug in a Drug Complex
Source: PLoS One. 2012 Mar 23;7(3):e33709. doi: 10.1371/journal.pone.0033709 (PMC3311648; doi:10.1371/journal.pone.0033709)

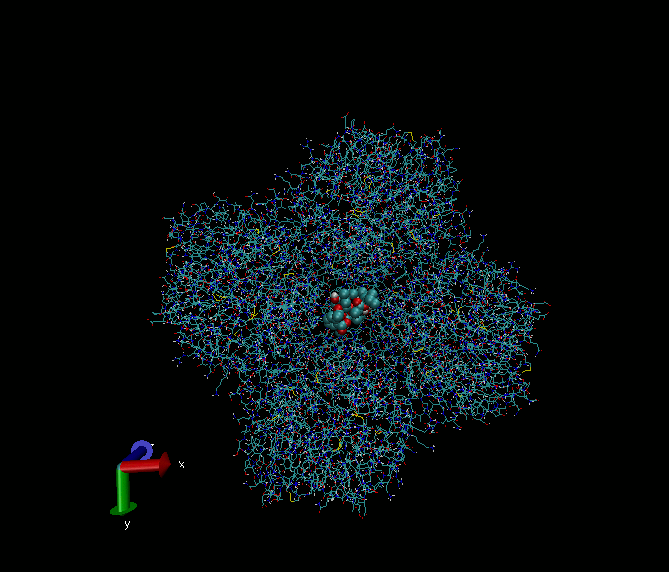

Supplement: Supporting Information S2 — The movie to show that Azithromycin & Aspirin complex cannot be separated from pocket_2hu4 when the elasticity coefficient of the spring is 1,000,000 kJ mol nm−2 (GIF) [file pone.0033709.s002.gif]
